# Mechanical Coupling between Endoderm Invagination and Axis Extension in *Drosophila*

**DOI:** 10.1371/journal.pbio.1002292

**Published:** 2015-11-06

**Authors:** Claire M. Lye, Guy B. Blanchard, Huw W. Naylor, Leila Muresan, Jan Huisken, Richard J. Adams, Bénédicte Sanson

**Affiliations:** 1 Department of Physiology, Development and Neuroscience, University of Cambridge, Cambridge, United Kingdom; 2 Cambridge Advanced Imaging Centre, University of Cambridge, Cambridge, United Kingdom; 3 Max Planck Institute of Molecular Cell Biology and Genetics, Dresden, Germany; The Francis Crick Institute, UNITED KINGDOM

## Abstract

How genetic programs generate cell-intrinsic forces to shape embryos is actively studied, but less so how tissue-scale physical forces impact morphogenesis. Here we address the role of the latter during axis extension, using *Drosophila* germband extension (GBE) as a model. We found previously that cells elongate in the anteroposterior (AP) axis in the extending germband, suggesting that an extrinsic tensile force contributed to body axis extension. Here we further characterized the AP cell elongation patterns during GBE, by tracking cells and quantifying their apical cell deformation over time. AP cell elongation forms a gradient culminating at the posterior of the embryo, consistent with an AP-oriented tensile force propagating from there. To identify the morphogenetic movements that could be the source of this extrinsic force, we mapped gastrulation movements temporally using light sheet microscopy to image whole *Drosophila* embryos. We found that both mesoderm and endoderm invaginations are synchronous with the onset of GBE. The AP cell elongation gradient remains when mesoderm invagination is blocked but is abolished in the absence of endoderm invagination. This suggested that endoderm invagination is the source of the tensile force. We next looked for evidence of this force in a simplified system without polarized cell intercalation, in acellular embryos. Using Particle Image Velocimetry, we identify posteriorwards Myosin II flows towards the presumptive posterior endoderm, which still undergoes apical constriction in acellular embryos as in wildtype. We probed this posterior region using laser ablation and showed that tension is increased in the AP orientation, compared to dorsoventral orientation or to either orientations more anteriorly in the embryo. We propose that apical constriction leading to endoderm invagination is the source of the extrinsic force contributing to germband extension. This highlights the importance of physical interactions between tissues during morphogenesis.

## Abstract 2

A study of the mechanism of axis extension in developing *Drosophila* embryos reveals that apical constriction leading to invagination of the posterior endoderm produces a tensile force that pulls the extending germband.

## Author Summary

Embryos change shape dramatically during development. The genetic programs that drive the active behavior of cells underlying these changes are well understood, but little is known about how movements of neighboring tissues influence the shaping of a given tissue. We address this question for the anteroposterior elongation of the body axis (germband) of *Drosophila* embryos. We had previously shown that during elongation, the germband cells stretch along the anteroposterior axis, in addition to undergoing active rearrangements; this suggested that extrinsic tensile forces might be at play. In the current study we find that the start of main body elongation is synchronous with the invagination of both the mesoderm and the endoderm. We analyze mutants and find that cell stretching disappears in embryos lacking endoderm invagination but remains in those lacking mesoderm invagination. We then measure tension using laser ablation in acellular embryos that lack active cell rearrangements in the germband but undergo the initial stages of endoderm invagination. We find that tension is higher in the anteroposterior direction close to the invaginating endoderm. Our results indicate that endoderm invagination generates a tensile force that is transmitted to the germband, and contributes to its elongation. This study reveals how tissues interact during embryo morphogenesis.

## Introduction

During development, many tissues extend in one orientation while narrowing in the orthogonal one. These so-called convergence and extension movements elongate the anteroposterior axis in bilateral animals during gastrulation, where they have been most studied [1–4]. Defects in convergence and extension movements at gastrulation have been linked to neural tube defects in mouse and human embryos [5]. Convergence and extension movements are also important later in embryo morphogenesis, for example for the elongation of the cochlear tube [6], the kidney tubules [7], and the limb and jaw cartilages [2].

Intracellular forces are key in convergence and extension and in most cases studied, drive polarized cell rearrangements [1,2]. These require planar polarization of proteins at cell membranes [3,8]. Planar polarization of actomyosin was first shown in *Drosophila* germband extension (GBE) to result in the selective shortening of dorsoventrally (DV) oriented cell contacts [9,10]. The cell biology of this process has since been extensively characterized, and planar polarization of several other components including Bazooka (the homologue of Par-3) and E-cadherin have been found to be required for active cell rearrangements [11–20]. These polarities are controlled by the anteroposterior (AP) segmentation cascade in *Drosophila*, the most downstream genes being the pair-rule genes, encoding transcription factors such as Even-skipped and Runt [9,10,21]. Recent work has found that a combinatorial code of Toll-like receptors is required for transducing the AP positional information from these transcription factors into the planar polarities required for polarized cell intercalation [22]. Recently, actomyosin-driven shortening of cell contacts has also been shown to be essential for convergence and extension movements in vertebrates [7, 23–25].

However, cell-autonomous behaviors might not be sufficient to fully explain axis elongation [26]. Stresses generated by neighboring morphogenetic movements or by the constrained geometry of the embryo could contribute to axis extension [27–29]. Evidence for extrinsic forces influencing tissue elongation has been reported: in *Caenorhabditis elegans*, body wall muscle contractions guide embryonic elongation [30]; in *Drosophila* oogenesis, the traction force produced by the follicle rotation is required for egg chamber elongation [31]; in the *Drosophila* developing wing, the contraction of the hinge produces a tensile stress that orients the cell behaviours required for wing blade elongation [32,33].

In the *Drosophila* embryo, we found previously that in addition to polarized cell intercalation, AP cell elongation contributes to GBE [34]. These cell shape changes are not a consequence of cell rearrangements: in the absence of polarized cell intercalation, the germband cells elongate even more in AP, a behavior most parsimoniously explained by an extrinsic tensile force acting on the tissue [34]. This gives us the opportunity to investigate how extrinsic factors can contribute to axis extension. Here, we search for the source of the extrinsic force acting on the germband by measuring the deformation of cells as a function of time, in the absence and presence of other morphogenetic movements. We find that blocking posterior endoderm invagination abolishes AP cell elongation. Furthermore, we present evidence that apical constriction leading to invagination of the posterior endoderm primordium produces a tensile force propagating from the posterior of the embryo. We conclude that this gastrulation movement at the posterior produces an AP tensile force contributing to the elongation of the main axis in *Drosophila*.

## Results

### The Patterns of AP Cell Elongation Form a Gradient Increasing Towards the Posterior Tip of the Embryo

**Fig 1:**
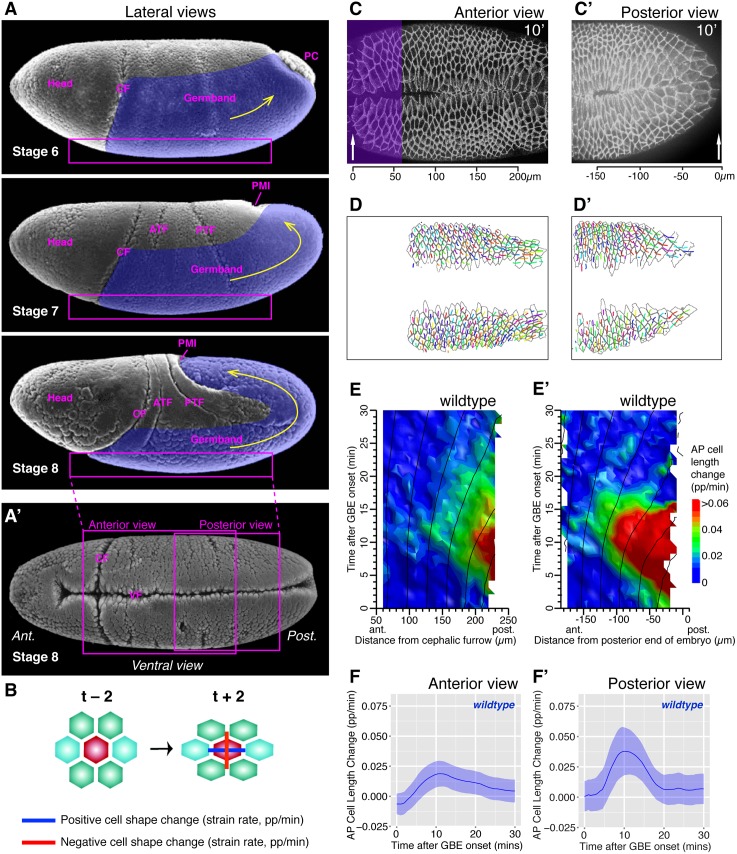
Cell shape change analysis during *Drosophila* axis extension. (A) Scanning electron microscopy micrographs (from Flybase, [36]) showing lateral views of gastrulating *Drosophila* embryos at stage six, seven, and eight. Anterior is to the left. The part of the germband undergoing convergent extension is labelled in purple and the main direction of extension indicated with a yellow arrow. The following landmarks or morphogenetic events are indicated: PC, pole cells; CF, cephalic furrow; ATF, anterior transverse furrow; PTF, posterior transverse furrow; PMI, posterior midgut invagination (also called posterior endoderm invagination). The box represents the ventral surface that we optically sectioned by confocal microscopy. (A’) Scanning electron microscopy micrograph showing a ventral view of a gastrulating embryo at stage eight, with the approximate position of the anterior and posterior field of views that we analyzed. Both views are bissected by the ventral furrow (VF) through which the mesoderm invaginates. (B) Schematics representing the small neighborhood of cells considered by the tracking algorithms. Cell shape changes (strain rates) are calculated by comparing two timepoints before and after a given time. A strain rate is the ratio of the change in length to the original length, divided by the time interval, with units of proportion per minute (pp/min). The cell shape change is represented here by two orthogonal vectors, showing elongation in one direction (blue, positive) and shrinkage in the perpendicular direction (red, negative). (C, C’) Example frames of movies of wild-type embryos at t = 10 min after GBE onset labeled with *ubi-DE-cad-GFP*, showing an anterior (C, wtLB009) and a posterior view (C’ wtCL051010). (C) For anterior views, the cephalic furrow (arrow) is used as an anterior landmark, and the scale shows distance from this landmark. The purple shading shows the region removed from the analysis, where cells stretch behind the cephalic furrow. (C’) For posterior views, the posterior-most edge of the embryo seen in the confocal stack is used as posterior landmark (arrow), with distance from it indicated in the scale. (D, D’) Outcome of tracking for anterior (D) and posterior views (D’), showing the polygons describing the cell outlines and the cell centroids from which are drawn the tracks giving the cell positions for the previous 2.5 min (5 timepoints). The tracks shown are track retained for the analysis, after removing tracks from mesodermal cells, mesectoderm cells, and for anterior views, from cells deformed by the cephalic furrow (corresponding to purple region in C). (E, E’) Spatiotemporal heat maps summarizing AP cell length change contributing to GBE, over the first 30 min of GBE (*y*-axis) and as a function of cell position in the AP axis (*x*-axis), for anterior (E) and posterior views (E’), averaged for five and four embryos, respectively. (F, F’) Graphs summarizing AP cell length change as a function of time for the first 30 min of GBE, for anterior (F) and posterior views (F’), averaged for five and four embryos, respectively. The ribbon’s width shows the standard error (see Materials and Methods). Data associated with this figure can be found in S1 Data.

We analyzed apical cell shape changes using custom-made algorithms as previously [34,35]. We imaged embryos labeled with the junctional marker *ubi-DE-cad-GFP* on their ventral side by confocal time-lapse microscopy, acquiring images every 30 s at 20.5 ± 1°C, starting movies around morphological stage six and finishing around stage eight (Fig 1A, 1A’, 1C and 1C’). We segmented apical cell contours based on the fluorescent signal and linked cells in time, storing the coordinates of the centroid of each cell and of a polygon describing its outline, at each timepoint (Fig 1D and 1D’). To measure the cell shape changes, our algorithms consider small cell neighborhoods consisting of a central cell surrounded by one ring of its immediate neighbors (Fig 1B). Cell shapes for this neighborhood are measured by fitting an ellipse to each cell: strain rates are calculated over a 2 min window (±2 timepoints, see Fig 1B). To analyze specifically the AP component of cell shape change (the component that will contribute to axis extension), the strain rates were projected onto the AP embryonic axis. In our summary plots, we call this strain rate “AP cell length change,” expressed in proportion per minute (pp/min) (Fig 1E–1F’) and shorten it to “AP cell elongation” in the text thereafter. Note that from our measures of strain rates, we can also extract DV cell elongation and cell area change (see below). To consider only the deformation of cells from the germband (the tissue undergoing convergence and extension), we excluded any tracks from mesoderm and mesectoderm cells (Fig 1D and 1D’). These methods allow us to examine the patterns of AP cell elongation in living embryos, which we proposed to be a signature of an extrinsic force contributing to axis extension [34].

We had previously analyzed AP cell elongation in field of views that included the cephalic furrow as an anterior landmark (the cephalic furrow forms between the head and the germband) [34] (Fig 1A, 1A’ and 1C). These views show the anteriormost region of the ventral side of the germband and are thereafter called “anterior views” for simplicity. When visualizing AP cell elongation as a function of time and position along the AP axis in spatiotemporal heat maps, we noticed that the signal was higher towards the posterior edge of the field of view [34] (average for five movies, Fig 1E; individual movies, S1A Fig; tracking information, S1C and S1C’ Fig). This prompted us to image the ventral side of embryos more posteriorly, using the tail end of the embryo (as detected in apical optical sections) as a posterior landmark (Fig 1A’ and 1C’). Plotting spatiotemporal maps for these “posterior views” revealed that AP cell elongation becomes even stronger closer to the posterior tip of the embryo (average for four movies, Fig 1E’; individual movies, S1B Fig; tracking information, S1D and S1D’ Fig; example S1 Movie). Indeed, although AP cell elongation peaks around 10 min after GBE onset in both views, the magnitude is doubled in posterior views: 0.04 pp/min (average for four movies, Fig 1F’) compared to 0.02 pp/min in anterior ones (average for five movies, Fig 1F). Note that to be able to make fair comparisons between anterior and posterior views, we removed the tracks of ectodermal cells deformed by the cephalic furrow in anterior views (purple shaded region in Fig 1C, resulting tracks in Fig 1D), since these unrelated cell deformations would otherwise contribute to our measure of total AP cell elongation, as they did in our previous study [34]. All anterior views presented in this paper have been reanalyzed with this exclusion. We estimated that in wild-type embryos, the two fields of view overlapped by about 80 microns (Fig 1A’), and we concluded that the patterns of AP cell elongation detected in posterior views fully included the patterns seen in anterior views (Fig 1E and 1E’).

**Fig 2:**
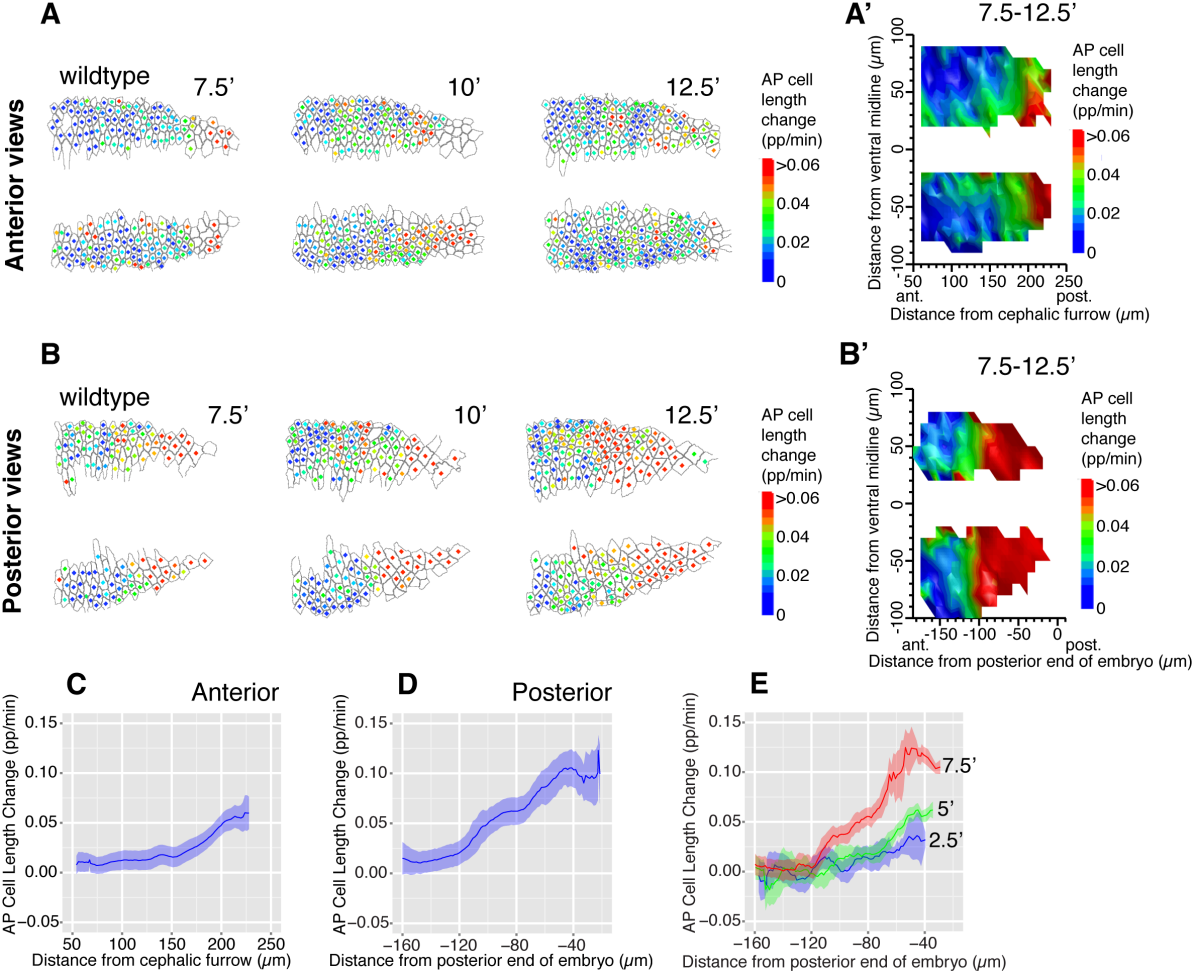
AP cell elongation patterns form an AP gradient. (A, B) AP cell length change shown for each analyzed cell, for timepoints 7.5, 10, and 12.5 min after GBE onset in anterior (A, wtLB009) and posterior views (B, wtCL051010). The color of the dot at the center of each cell corresponds to the scale bar shown. (A’, B’) Spatial maps summarizing AP cell length change over the 7.5–12.5 min time interval, as a function of the position of cells in the AP (*x*-axis) and DV (*y*-axis) embryonic axes, for anterior (A’) and posterior views (B’) (average for five and four embryos per views, respectively). (C, D) Graphs summarizing AP cell length change over the 7.5–12.5 min time interval, as a function of cell position in the AP axis, for anterior (C) and posterior views (D). (E) Graphs summarizing AP cell length change (*y*-axis), at 2.5, 5, and 7.5 min after the onset of GBE, as a function of cell position in the AP axis (*x*-axis) for posterior views (average for four wild-type embryos). The ribbon’s width shows the standard error (see Materials and Methods). Data associated with this figure can be found in S2 Data.

The AP cell elongation patterns appeared to form a gradient increasing from the anterior to the posterior. To ascertain this, we examined a short period around the peak of AP cell elongation, from 7.5 min to 12.5 min after GBE onset (Fig 2). This confirmed that AP cell elongation increased steeply towards the posterior of the embryo (Fig 2A–2D), forming a gradient over a distance of about 150 μm in posterior views (average for four movies, Fig 2D). Although the gradient is clearest in posterior views, some AP gradation was already detectable in anterior views (average for five movies, Fig 2C), consistent with the notion that we are visualizing the beginning of the gradient in anterior views. In posterior views, we also looked at snapshots of the gradient earlier in GBE, at 2.5, 5, and 7.5 min: the gradient was at first shallow and confined to the more posterior part of the field of view; it then expanded towards the anterior and became steeper with time (Fig 2E). These results suggested that a tensile stress deformed the tissue from a posterior source, starting at the onset of GBE and propagating towards the anterior of the embryo over time.

We also analyzed cell area change in addition to AP cell elongation (S2A and S2A’ Fig). When passively responding to planar extrinsic forces, cell apical areas are expected to change in opposite ways depending on whether cells are compressed or pulled: when pulled, cell areas should increase; in contrast, when compressed, cell areas should decrease. We had already noted in our previous study that AP cell elongation was accompanied by an increase in cell area in anterior views, supporting the idea that the germband was experiencing a planar tensile stress [34] (S2A Fig). This trend is even clearer for the posterior views: the patterns of AP cell elongation are matched by patterns of cell area increase, suggesting that the germband cells elongated in response to a tensile rather than compressive stress (compare S2A’ Fig with Fig 1E’). Note that in our analyses, we can observe changes in only the two planar axes defining the apical cell areas, but we expect the third axis, the cell length in Z, to increase or decrease in response to planar stress to keep the cell volume constant [37,38].

Around the onset of GBE, the germband cells are also subjected to a pull in the perpendicular direction, along DV, in response to the invagination of the mesoderm on the ventral side of the embryo [34] (mesoderm invaginates through a ventral furrow visible in Fig 1A’, 1C and 1C’). In both anterior and posterior views, we found that DV elongation of ectodermal cells in response to mesoderm invagination have patterns completely distinct from the AP cell elongation patterns that we are focusing on in this study: first, they are most prominent close to GBE onset and have disappeared by 10 min into GBE (whereas the AP cell elongation patterns peak just after 10 min), and second, they occur uniformly along the AP axis of the embryo (whereas the AP cell elongation patterns occur in a posterior gradient) (S2B–S2C” Fig). Note that AP and DV cell elongation patterns are both accompanied by an increase in cell area (S2A and S2A’ Fig), consistent with the idea that they are both the consequence of tensile forces. We concluded that germband cells are subjected to two independent tensile forces, one in the DV direction caused by mesoderm invagination (see also below), and one in the AP direction coming from the posterior of the embryo.

Together, our analysis of wild-type *Drosophila* embryos indicated that AP cell elongation formed an AP gradient consistent with a stress propagating from the posterior. We asked next what the origin of this tensile force was.

### The Gradient of AP Cell Elongation during Axis Extension Is Still Present in Absence of Mesoderm Invagination

**Fig 3:**
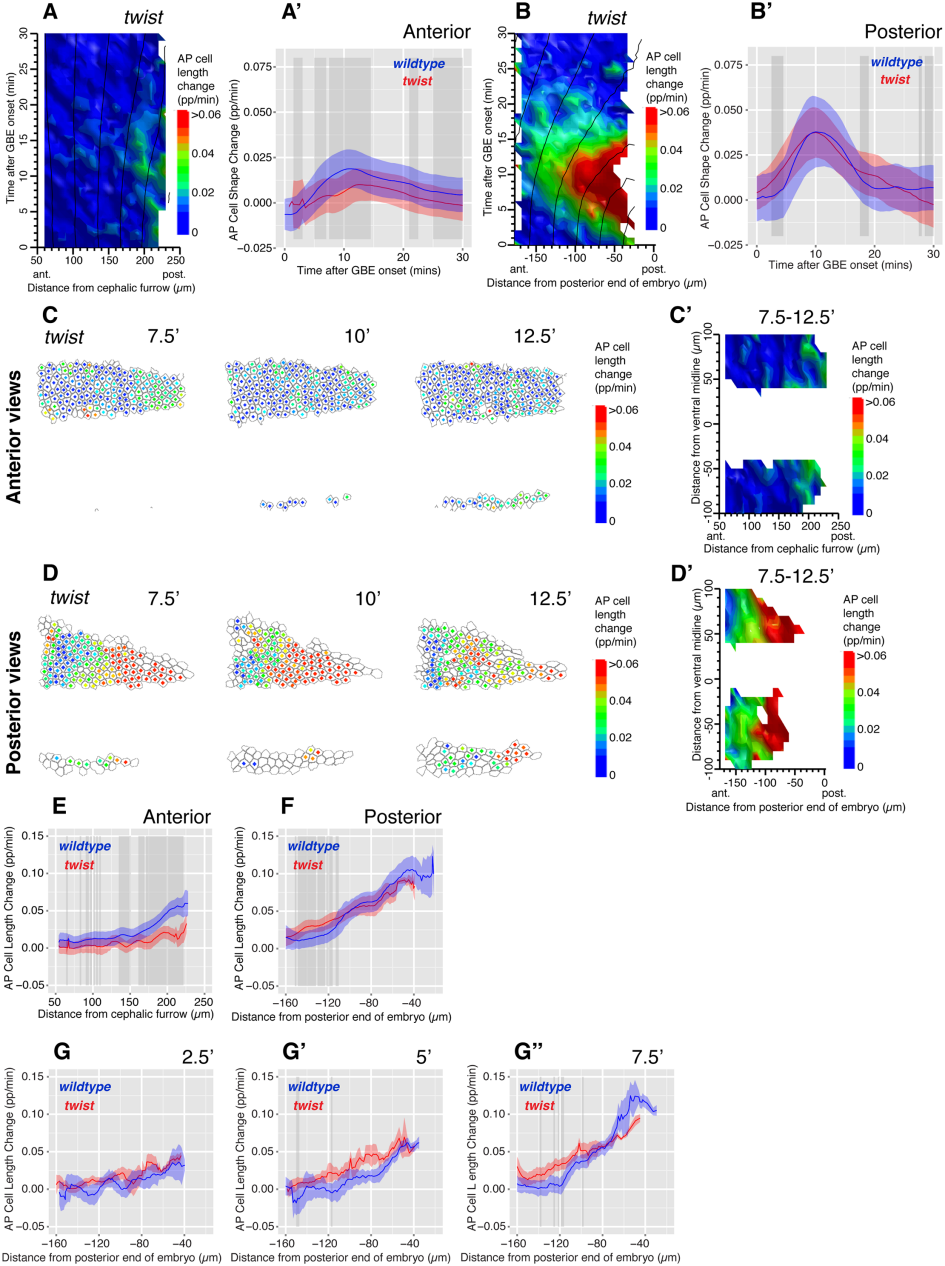
The AP cell elongation gradient is present in *twi* mutant embryos. (A) Spatiotemporal map summarizing AP cell length change contributing to GBE, over the first 30 mins of GBE (*y*-axis), and as a function of cell position in the AP axis (*x*-axis), for *twi* mutants in anterior views (average for five embryos). (A’) Graph comparing AP cell length change as a function of time for the first 30 min of GBE, in wild-type (blue) and *twi* mutants (red) for anterior views (average for five embryos each). In these graphs and thereafter, the ribbon’s width indicates the standard error, and the grey-shaded boxes show where a difference is statistically significant (*p* < 0.05, see Materials and Methods). (B, B’) Equivalent plots as A, A’ for posterior views. (C, D) AP cell length change shown for each analyzed cell, for timepoints 7.5, 10, and 12.5 min after GBE onset in *twi* mutant embryos, for movie frames of an anterior (C, twiLB012) and a posterior view (D, twiCL140411). The color of the dot at the center of each cell corresponds to the scale bar shown. (C’, D’) Spatial maps summarizing AP cell length change over the 7.5–12.5 mins time interval, as a function of the position of cells in the AP (*x*-axis) and DV (*y*-axis) embryonic axes, for anterior and posterior views in *twi* mutants (average of five and three embryos per view, respectively). (E, F) Graphs comparing AP cell length change over the 7.5–12.5 min time interval, as a function of cell position in the AP axis, for wild-type (blue) and *twi* mutant (red) embryos, for anterior and posterior views. (G–G”) Graphs summarizing AP length change (*y*-axis), at 2.5, 5, and 7.5 min after the onset of GBE, as a function of cell position in the AP axis (*x*-axis) for wild-type (blue) and *twi* mutant (red) embryos, for posterior views (average for four and three embryos per genotype, respectively). Data associated with this figure can be found in S3 Data.

A stress propagating from the posterior seemed at odds with our previous model suggesting a role for mesoderm invagination in generating AP patterns of cell elongation [34]. This model was based on the analysis of anterior views, where we had previously found that AP cell elongation contributing to axis extension was reduced in *twist* (*twi*) mutants, which are defective for mesoderm invagination. Although we had proposed at the time that mesoderm invagination might contribute to the extrinsic tensile force deforming the germband, it was difficult to formulate a model for how it could do so [29,34]. We reanalyzed the data from anterior views after exclusion of the region deformed by the cephalic furrow (see above). We confirmed our previous results: in anterior views, AP cell elongation was significantly reduced in *twi* mutants compared to wild type (average for five movies, Fig 3A and 3A’; individual movies, S3A Fig; example S2 Movie). Next, we acquired new movies imaging the posterior ventral side of the embryo, using the posterior end of the imaged embryo as a landmark, as before for wild type. To our surprise, we found robust AP cell elongation in posterior views of *twi* embryos, with no statistical difference between the rate of AP cell length change between these mutant embryos and wild type (average for three movies, Fig 3B and 3B’; individual movies, S3B Fig). Elongating cells tended to increase in area in these posterior views, suggesting that they elongated in response to a tensile stress, as in wild type (S3C’ Fig). Note that in these cell area plots, the cell area increase in response to mesoderm invagination is absent (0 to 5–7 min), in posterior as in anterior views, demonstrating that the embryos we imaged are indeed defective for mesoderm invagination (compare S2A Fig with S3C Fig, and S2A’ Fig with S3C’ Fig). Further demonstrating this, DV cell elongation is gone in anterior and posterior views of *twi* mutant embryos (S3D, S3D”, S3E and S3E” Fig, compare with S2B, S2B”, S2C and S2C” Fig). This shows that whereas the early DV stretch of ectodermal cells is gone as expected in *twi* mutants (because there is no mesoderm invagination to pull the ectoderm in DV), the AP stretch of ectodermal cells is still present in posterior views (S3E and S3E’ Fig). This confirmed that DV and AP cell elongation were produced by two independent tensile forces, and that mesoderm invagination caused DV cell elongation in the germband. Refuting our previous model [34], this also indicated that mesoderm invagination did not cause the AP cell elongation contributing to GBE.

As before for wild type, we examined the gradient of AP cell elongation between 7.5 and 12.5 mins and confirmed that there is a significant difference with wild type for anterior views but no clear statistical difference when comparing posterior views (Fig 3C–3G”). This discrepancy suggested that the relative position of anterior and posterior fields of view are different in wild-type and *twi* mutants, leading to the detection of the AP cell elongation gradient in posterior views, but not in anterior views, in *twi* mutants. This is likely to be the result of several factors, one of which might be a difference in curvature on the ventral side of the embryo between the two genotypes. Indeed, we find that the outlines of *twi* embryos are less curved than wild-type ones in anterior views, and the embryos are wider, consistent with the notion that *twi* embryos are flatter (S4 Fig). A flatter ventral surface in *twi* mutants would make the posterior views more posteriorly located in *twi* mutants, because the position of the posterior landmark we use (the tip of the embryo in optical sections) will be influenced by curvature. A flatter surface could be a direct consequence of the failure of mesoderm invagination and the absence of a keel-like shape in *twi* mutants. Absence of invaginating mesoderm could not only affect the curvature of the embryo, but also change its mechanical properties and, for example, make it flatten more under a coverslip during imaging. Both factors would make the anterior and posterior fields of view further apart in *twi* mutants compared to wild type.

We concluded that a gradient of AP cell elongation was present in *twi* mutants and grossly similar to wild type in posterior views, showing that an event other than mesoderm invagination must be responsible for the AP extrinsic force deforming the germband.

### The Start of Posterior Endoderm Invagination Is Synchronous with GBE Onset

**Fig 4:**
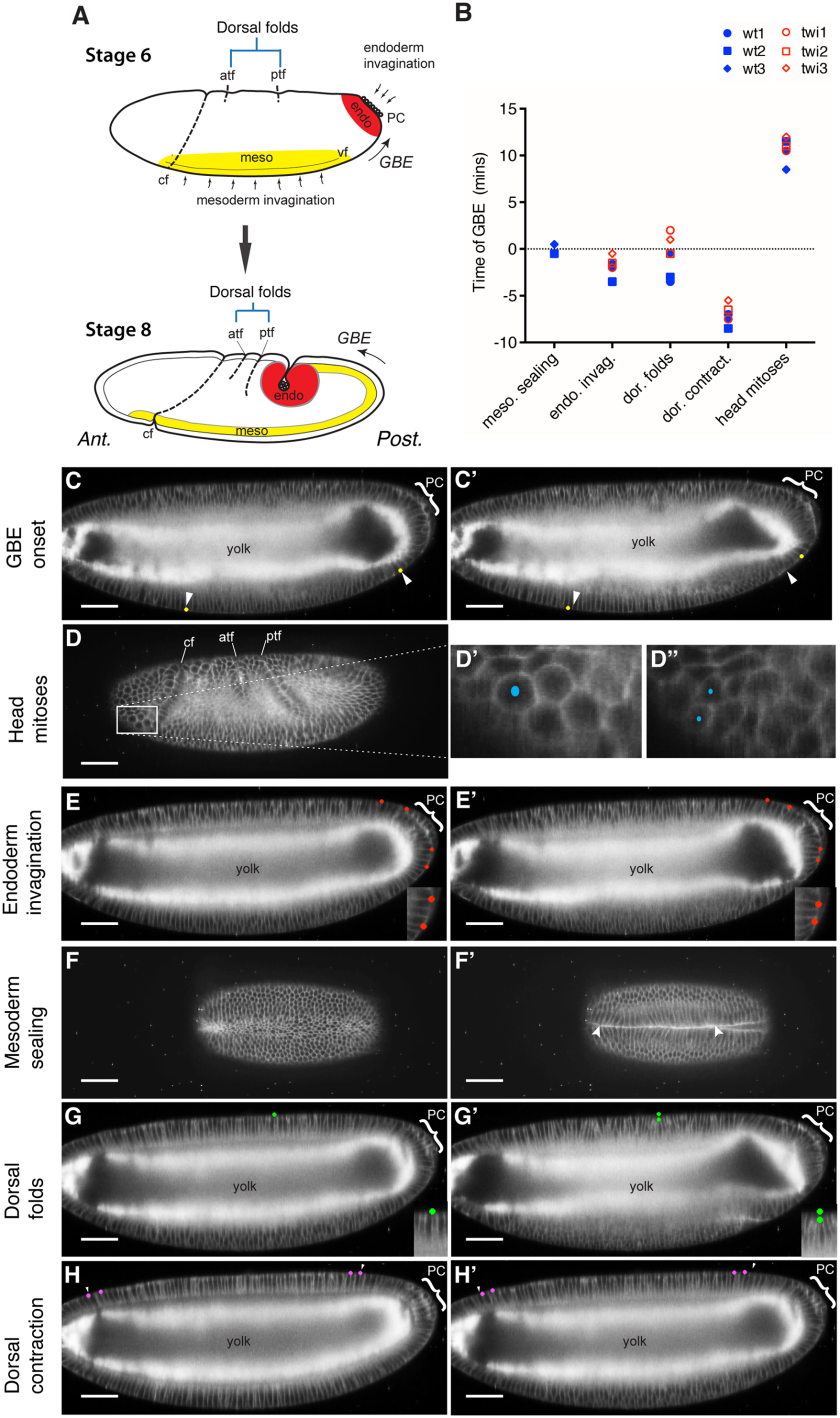
Temporal relationships between morphogenetic movements during *Drosophila* gastrulation. (A) Diagram showing the sites of the different morphogenetic movements on lateral views of stage six and eight embryos (see also Fig 1A and 1A’). The invaginating mesoderm and endoderm layers are shown in yellow and red, respectively. At stage six, these layers are in the process of invagination from the surface to the interior of the embryo, and by stage eight, both of these layers are fully internalized. PC, pole cells; CF, cephalic furrow; ATF, anterior transverse furrow; PTF, posterior transverse furrow; GBE, germband extension. (B) Graph summarizing the temporal mapping of the head mitoses and four morphogenetic events relative to the onset of GBE, for three wild-type and three *twi* mutant movies. (C–H’) All views are taken from a wild-type embryo labeled with the whole-membrane markers *resille-GFP* and *spider-GFP* and imaged by light sheet microscopy (SPIM). For each morphogenetic movement, two movie frames are shown, before and after the temporally mapped event. The position of the pole cells (PC) at the posterior of the embryos are indicated by a bracket. All embryos shown are at either late stage five or early stage six, except the embryo in D, which is at stage eight. At these early stages, embryonic cells are arranged in a single columnar layer surrounding a large yolk cytoplasm, visible as a bright signal in lateral views. (C, C’) Lateral views showing the mapping of individual cells (yellow dots) 1 min before (C) and 1.5 min after (C’) the onset of GBE. The arrowheads indicate the position of the cells before the start of GBE. (D–D”) Lateral views with a box highlighting the mitotic domain 2 in the head [40], with close-up in (D’) showing a cell about to divide and (D”) the two resulting daughter cells 3.5 min later. (E, E’) Lateral views showing the mapping of cells (red dots) 2 min before (E) and 2 min after (E’) the onset of apical constriction in the endoderm primordium. The close-ups show that the cells between the pair of red dots constricted their apices, causing the dots to move closer together. (F, F’) Ventral view showing the ventral furrow 2.5 min before (F) and 1.5 min after (F’) mesoderm sealing. (G, G’) Lateral view showing the mapping of the apical surface of a cell in comparison to its original position (green dots) on the dorsal surface 2.5 min before (G) and 4 min after (G’) the initiation of the posterior transverse fold (one of the two dorsal folds). The close-up shows the basalwards displacement of the apical surface. (H, H’) Lateral view showing the movement of individual cells (magenta dots) 1 min before (H) and 3 min after (H’) dorsal contraction. The arrowheads show the cells’ positions before contraction. Data associated with this figure can be found in S4 Data.

We reasoned that candidates for generating a tensile stress at the posterior would be morphogenetic movements taking place at, or just before, the onset of GBE, because germband cells start to elongate in AP from the beginning of GBE [34] (Fig 1E and 1E’). To identify such events, we measured the timings of gastrulation movements relative to the start of GBE (Fig 4). Because some movements take place on the ventral surface (mesoderm invagination) and others on the dorsal surface (endoderm invagination, dorsal folding) (Fig 4A, see also Fig 1A and 1A’), we used light sheet microscopy (SPIM, selective plane illumination microscopy) to image the whole embryo volume through developmental time [39]. We labelled the cells with plasma membrane markers such as *Spider-GFP* and *Resille-GFP* and took timepoints every 30 sec (at 28–30°C). We examined three wild-type movies and three *twi* mutants defective for mesoderm invagination (Fig 4B).

We mapped the onset of GBE by identifying the first posteriorward displacement of ventral cells (Fig 4C and 4C’, and S3 Movie) and used the corresponding time-point as time zero for all the movies. To check that the development rates of all embryos imaged were comparable, we used patterned mitoses in the head as a developmental timer (Fig 4D– 4D”)[40]. We found that these mitoses start at 8.5 min, 10.5 min, and 11.5 min after GBE onset in the three wild-type movies and at 10.5 min, 11 min, and 12 min in the three *twi* movies (Fig 4B). This showed that there were no obvious differences in development rates between embryos and illustrates the temporal reproducibility of *Drosophila* early development.

Next, we mapped the timings of morphogenetic movements visible in the movies (Fig 4A) (for a review of the anatomy of these movements, see [29]). We concluded that the two morphogenetic movements most synchronous with GBE onset were mesoderm and posterior endoderm invaginations (Fig 4B). We mapped the onset of posterior endoderm invagination (also called posterior midgut invagination) by identifying in which movie frame the cells initiated apical constriction at the posterior of the embryo (Fig 1E and 1E’ and S3 Movie). Posterior midgut invagination preceded GBE by −3.5, −2, and −1.5 min in the three wild type, and by −2, −1.5, and −0.5 min in the three *twi* mutant embryos (Fig 4B). To map a clearly identifiable step of mesoderm invagination, we recorded the timepoint when the right and left sides of the mesoderm first met to begin forming the internal mesodermal tube, thereafter called “mesoderm sealing” (Fig 4F and 4F’ and S4 Movie). The times relative to the onset of axis extension were −0.5 min, −0.5 min, and +0.5 min for the 3 wild type movies (*twi* embryos fail to form a mesodermal tube) (Fig 4B). This confirms a remarkable synchrony between mesoderm sealing and GBE onset, which we had noted before [34] (see Discussion). We also looked at morphogenetic movements that occur on the dorsal side of the embryo. Dorsal folding occurs in two stereotyped locations under the control of the AP patterning system [41]. Although these folds start forming just before the onset of axis extension in wild type embryos, they initiate after GBE onset in two out of three *twi* embryos (Fig 4G and 4G’). Since AP cell elongation at the posterior end of the embryo are already high in *twi* mutants at GBE onset (Fig 3B), this suggests that the dorsal folds are not initiating these (although they could later contribute). Other dorsal movements include a dorsal contraction (Fig 4H, 4H’ and 4B) and the onset of amnioserosa cell flattening [42]. These occur respectively too early and too late, relative to the onset of GBE, to be key influences.

We conclude from this temporal mapping of morphogenetic movements that both mesoderm sealing and endoderm invagination are synchronous with the onset of GBE. Since we have refuted a role of mesoderm invagination in producing the gradient of AP cell elongation contributing to axis extension (see previous section), posterior endoderm invagination was the main candidate to generate a tensile stress during GBE.

### The Gradient of AP Cell Elongation Requires an Intact Posterior Endoderm Invagination

To test a role of posterior endoderm invagination in AP cell elongation during axis extension, we examined *folded gastrulation* (*fog*) and *torso-like* (*tsl*) mutants that abolish endoderm invagination. *Fog* is a zygotic gene required for the apical constriction of the endoderm primordium cells arranged in a disc at the posterior, which leads to posterior midgut invagination [43]. The expression of *fog* in the posterior midgut primordium requires the zygotic gap genes *huckebein* and *tailless*, which themselves require the activity of the maternal gene *tsl*, an upstream component of the terminal patterning system [44]. In anterior views, no obvious AP cell elongation gradient was detected at the onset of GBE in *fog* mutants (compare S5B Fig with Fig 1E). However, *fog* mutants proved problematic to analyze because their extending germband form ectopic folds (arrows in S5A and S5B Fig and S7 Movie). These folds occur because the posterior end of the germband does not move away in these mutants, but polarized active cell intercalations still elongate the germband [43]. Folding stretched the germ-band cells locally and produced strong AP cell elongation, as seen on spatiotemporal maps from approximately 7 min after GBE onset (S5B Fig). As a consequence, the total AP cell elongation could not be meaningfully compared between wild-type and *fog* mutants.

**Fig 5:**
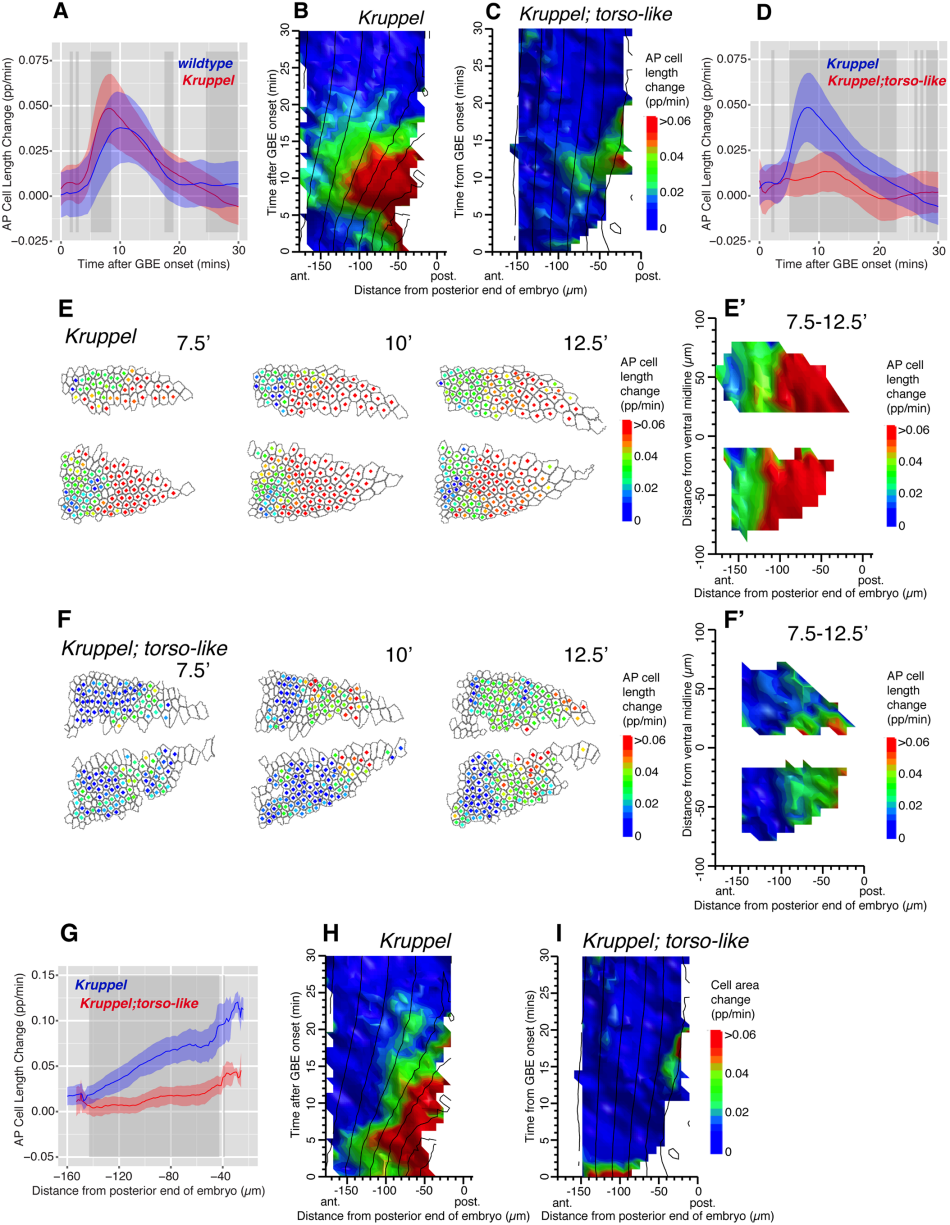
The AP cell elongation gradient is present in *Kr* but not *Kr; tsl* double mutants. (A) Graph comparing AP cell length change contributing to GBE as a function of time for the first 30 min of GBE, in wild-type (blue) and *Kr* mutants (red), in posterior views (average for four wild-type and three *Kr* embryos). In this graph and thereafter, the ribbon’s width indicates the standard error and the grey-shaded boxes show where a difference is statistically significant (*p* < 0.05, see Materials and Methods). (B, C) Spatiotemporal maps summarizing AP cell length change over the first 30 min of GBE (*y*-axis) and as a function of cell position in the AP axis (*x*-axis), for *Kr* and *Kr; tsl* mutants (average for three embryos of each genotype; individual movie plots in S5 Fig). (D) Graph summarizing AP cell length change as a function of time for the first 30 min of GBE for *Kr* (blue) and *Kr; tsl* mutants (red). (E, F) AP cell length change shown for each analyzed cell for timepoints 7.5, 10, and 12.5 min after GBE onset in a *Kr* (*KrCL051112*) and *Kr; tsl* mutant embryo (*KrtslCL040713*). The color of the dot at the center of each cell corresponds to the scale bar shown. (E’, F’) Spatial maps summarizing AP cell length change over the 7.5–12.5 min time interval, as a function of the position of cells in the AP (*x*-axis) and DV (*y*-axis) embryonic axes (average for three embryos per genotype). (G) Graph comparing AP cell length change over the 7.5–12.5 min time interval, as a function of cell position in the AP axis for *Kr* (blue) and *Kr; tsl* mutant (red) embryos (average for three embryos per genotype). (H, I) Spatiotemporal maps summarizing cell area changes with same axes as B, C for *Kr* and *Kr; tsl* mutant embryos. Data associated with this figure can be found in S5 Data.

To prevent folding, we analyzed one of the mutants that abolishes posterior midgut invagination, *tsl*, in combination with a mutant abolishing most of the active polarized cell intercalations in the trunk, *Kruppel* (*Kr*) [21,34]. To ask if *tsl* was required for the gradient of AP cell elongation, we compared *Kr* single mutants with these *Kr; tsl* double mutants. In posterior fields of views, AP cell elongations are slightly higher in *Kr* compared to wild type (Fig 5A). This was expected, since AP cell elongation increases in the absence of cell intercalation, presumably because in wild type, polarized cell intercalation acts to release some of the tensile stress in the germband [34]. The patterns of AP cell shape changes are, however, comparable in both genotypes (average for three movies, Fig 5B, compare with Fig 1E’; individual movies S5C Fig; example S5 Movie). As in wild type, the AP cell shape changes are accompanied by an increase in cell area, consistent with a tensile rather than compressive stress (Fig 5H; individual movies in S5D Fig). In double mutants *Kr; tsl* however, very little AP cell length change was detected (average for three movies, Fig 5C and 5D; individual movies S5E Fig; example S6 Movie). Note that the residual AP cell length change detected on the averaged spatiotemporal map (Fig 5C) was mainly present in one of the three individual movies (krtslCL040713, S5E Fig), and this signal was not accompanied by an increase in cell area, as would be expected for a tissue under tensile stress (S5F Fig). Consistent with this, there was no significant increase in cell area detected in double mutants *Kr; tsl* in the other two movies or in the averaged data (S5 Fig and Fig 5I). This indicated that the ectodermal cells in the posterior region of *Kr; tsl* mutants embryos were not under tensile stress.

We also examined in more detail the AP cell elongation gradient around its peak (from 7.5 to 12.5 min), in *Kr* versus *Kr; tsl* mutants. The steep gradient of AP cell elongation was abolished in *Kr; tsl* mutants (Fig 5E–5G). We concluded that posterior midgut invagination was required for the AP cell elongation contributing to axis extension in *Drosophila*.

### Constriction of the Apical Surface of the Posterior Endoderm Primordium in Acellular Embryos Produces an AP Tensile Stress

**Fig 6:**
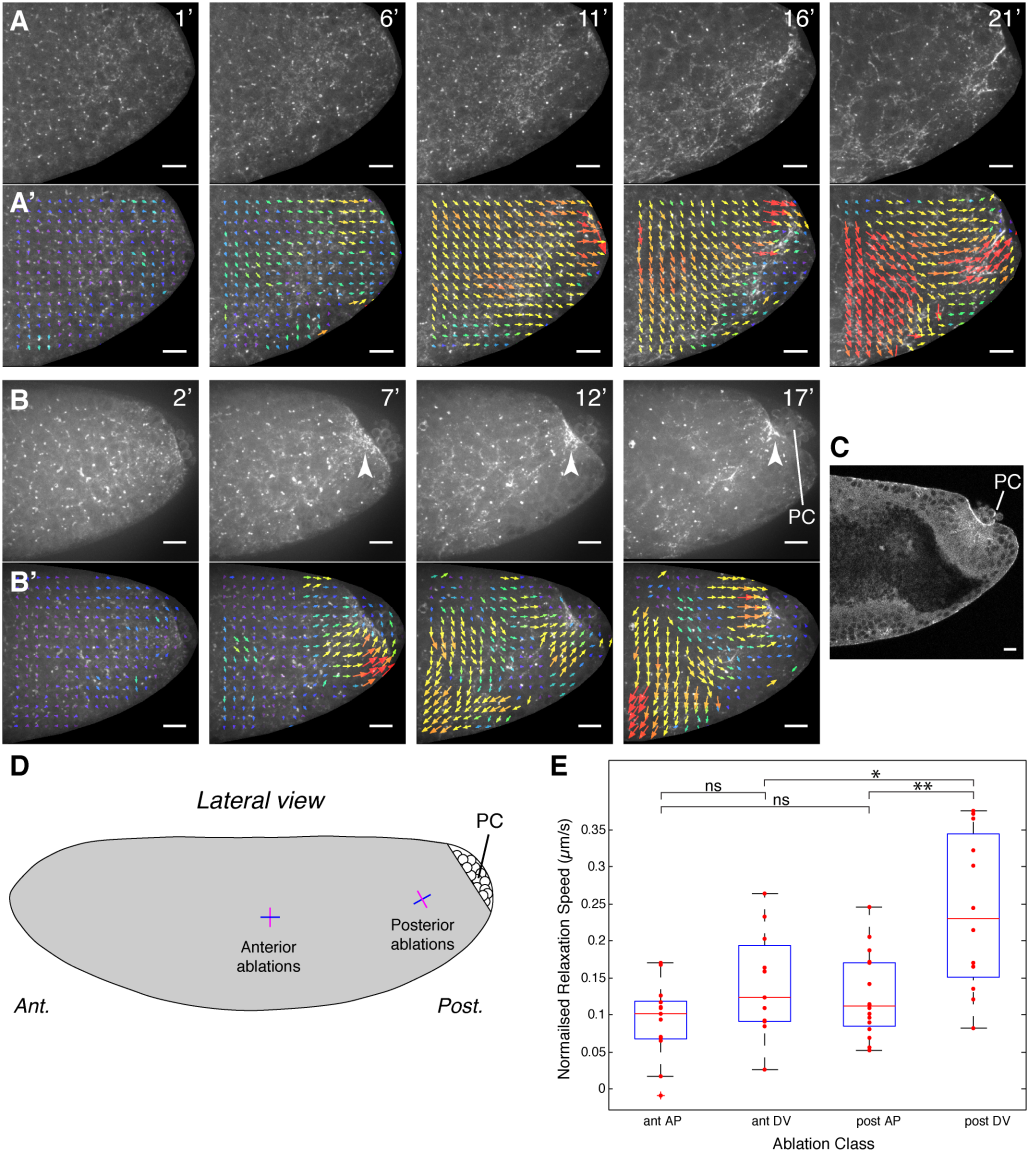
Apical constriction of the posterior endoderm primordium generates a tensile stress in acellular embryos. (A, B) Examples of movies of the posterior lateral surface of acellular embryos, with the actomyosin cytoskeleton labelled with *sqh-GFP* (see also S8 Movie, from which example A is taken). The Myosin II signal forms a disorganized meshwork at the apical surface of the embryo, which concentrates in the region close to the PC (see arrows in B). Occasionally, the meshwork becomes more cable-like, orienting towards the presumptive posterior endoderm (identified by the position of the PC). (A’, B’) Particle Imaging Velocimetry (PIV) tracking of the Myosin II signal reveals flows towards the presumptive posterior endoderm. Note that the ventralward flows also seen here move towards the ventral presumptive mesoderm (see S8 Movie). Arrows represent the displacement from the previous timepoint, scaled by a factor of four. Magnitude is shown using a heat scale, with fastest flows in red. Times shown are from the start of the movies. Scale bars are 20 microns. (C) Cross section of the posterior of an acellular embryo stained for Myosin II (see S6 Fig), showing the concentration of Myosin II where the apical surface has contracted and the beginning of an invagination. PC are indicated. (D) Schematics showing the position of the laser cuts performed on the lateral surface of the presumptive germband in acellular embryos (approximately to scale). The cuts are along a line 20 microns long, positioned either at the anterior (ant.) or at the posterior (post.) of the embryo, either orthogonal to the posterior flows (magenta, called DV thereafter for simplicity) or parallel to them (blue, called AP thereafter). (E) Dot plot with box plot overlaid showing the normalized relaxation velocities (corrected for displacement) for each category of cuts (*n* = 13 for ant. AP; n = 11, ant. DV; *n* = 16, post. AP; *n* = 12, post. DV). A two sample *t* test was used for the statistics (comparison ant. AP and ant. DV, ns: *p* = 0.070; comparison ant. AP and post. AP, ns: *p* = 0.1225; *: *p* = 0.0173; **: *p* = 0.0011). For the box plots, the central red line is the median, the edges of the box are the 25th and 75th percentile, the whiskers extend to the most extreme points not considered outliers, and outliers are plotted individually (Data points considered outliers are those more than 2.7 standard deviations from the mean). Data associated with this Figure can be found in S6 Data.

To understand more precisely how posterior endoderm invagination could produce a stress that in turn leads to a gradient of AP cell elongation, we analyzed a simplified system, in the form of acellular mutant embryos. Several mutations are known that produce embryos, which fail to cellularize. In one such mutant, an endoderm-like invagination is still visible on the dorsal side of the embryo, suggesting that apical constriction of the endoderm primordium still occurs in acellular embryos [45]. Consistent with this notion, another acellular mutant was shown recently to undergo apical constriction of the mesoderm primordium, albeit at a slower rate (about 60% of the wild type) [46]. To confirm that apical constriction also happened for the endoderm primordium, we made movies of these acellular mutants expressing *sqh-GFP* [46], to visualize the actomyosin cytoskeleton (*sqh* encodes the non-muscle Myosin II Regulatory Light Chain) (S8 Movie). We observed a concentration of Myosin II in the region where apical constriction would normally occur in the presumptive posterior endoderm, close to where the pole cells (PC) are attached, in both live and fixed embryos (Fig 6A and 6B, and S6D’ Fig). We find that the acellular embryos go through the initial steps of wild-type endoderm invagination [43], with first the formation of a flattened plate at the posterior (S6D’ Fig), then constriction of the embryo’s surface leading to some degree of invagination (Fig 6A–6C) (see also Fig 3a in [46]).

In live embryos, we noticed that the concentration of Myosin II at the posterior is accompanied by flows of Myosin II towards it (S8 Movie, top panel). This suggested that apical constriction of the presumptive endoderm surface could exert a tensile stress on the surrounding apical surface of the embryo. We also saw flows towards the ventral region, presumably in response to apical constriction of the presumptive mesoderm. We confirmed the direction of these flows by tracking the Myosin II signal at the surface of the acellular embryos using Particle Imaging Velocimetry (PIV). In our example movie showing the whole lateral surface of the presumptive germband, we can clearly see by PIV both ventralward (towards mesoderm) and posteriorward (towards posterior endoderm) flows of Myosin II signal (S8 Movie, bottom panel). To confirm the existence of posterior flows, we acquired more movies of the posterior end of the embryo and visualized the flows by PIV. We found that all embryos analyzed showed posteriorward flows towards the presumptive posterior endoderm (*n* = 8, 2 examples in Fig 6A’–6B’).

To understand better how the Myosin II flows relate to the surface membranes of the acellular embryos, we compared the localization of Myosin II with those of the E-cadherin complexes. Just before gastrulation movements started, E-cadherin and Myosin II colocalized in a hexagonal-like pattern (estimated stage 5; S6A, S6A’, S6C and S6C’ Fig). These presumably correspond to the regions of the surface membrane that, in wild-type embryos, would normally invaginate and become furrow canals encircling each syncytial nucleus (for example, see [47]). Once gastrulation movements started in acellular embryos, this relatively regular organization became disrupted: E-cadherin and Myosin II still colocalized but now formed a meshwork at the surface of the embryo (estimated stage 7; S6B, S6B’, S6D and S6D’ Fig). Since E-cadherin complexes are presumably associated with membranes, we infer that Myosin II flows that we observe track the movement of surface membranes in these embryos.

The presence of posteriorward flows of Myosin II signal in acellular embryos suggested that apical constriction of the presumptive endoderm was able to pull the apical surface behind it and could generate an AP tensile stress, which in wild-type embryos could contribute to axis extension. We reasoned that acellular embryos provided an excellent system in which to physically probe this tension, since it is unlikely to exhibit more complex morphogenetic behaviours such as polarized cell intercalation, and so we could rule out a contribution of the latter to measured tensions. No planar polarization of Myosin II was recognizable in these embryos, confirming that the apical surface of the embryo was unlikely to undergo intercalation-like movements (S6A–S6D’ Fig).

To directly test our hypothesis that apical constriction of the endoderm primordium generated a tensile stress at the posterior, we carried out line ablations at the surface of the embryo. Using a near-infrared laser, we made 20 micron-long incisions oriented parallel to the AP or DV embryonic axes and at the posterior or the anterior of the presumptive germband, on the lateral side of *sqh-GFP*-labelled acellular embryos (Fig 6D and S6E Fig). If, as we proposed, a tensile stress propagated from the posterior endoderm, we predicted that the DV cuts at the posterior should show a faster relaxation than any of the other three types of cuts. We used fine-grained PIV to track the movement of the Myosin II network, as a proxy for surface motion, and measured the velocities of recoil in a small region around the cuts, subtracting the velocity of that region before the cut to correct for translation (see Materials and Methods) (S6F–S6G’ Fig). We found that, as predicted, the average relaxation velocity of the DV cuts at the posterior was significantly higher than for any of the other cuts (Fig 6E). At the posterior, there was a clear anisotropy in the relaxation velocities, the DV-oriented cuts relaxing much faster than the AP-oriented cuts, whereas at the anterior, there was no statistically significant anisotropy. This provided evidence for an increased AP-oriented tension at the posterior of the embryo, in response to apical constriction leading to invagination of the endoderm primordium in acellular embryos.

## Discussion

**Fig 7:**
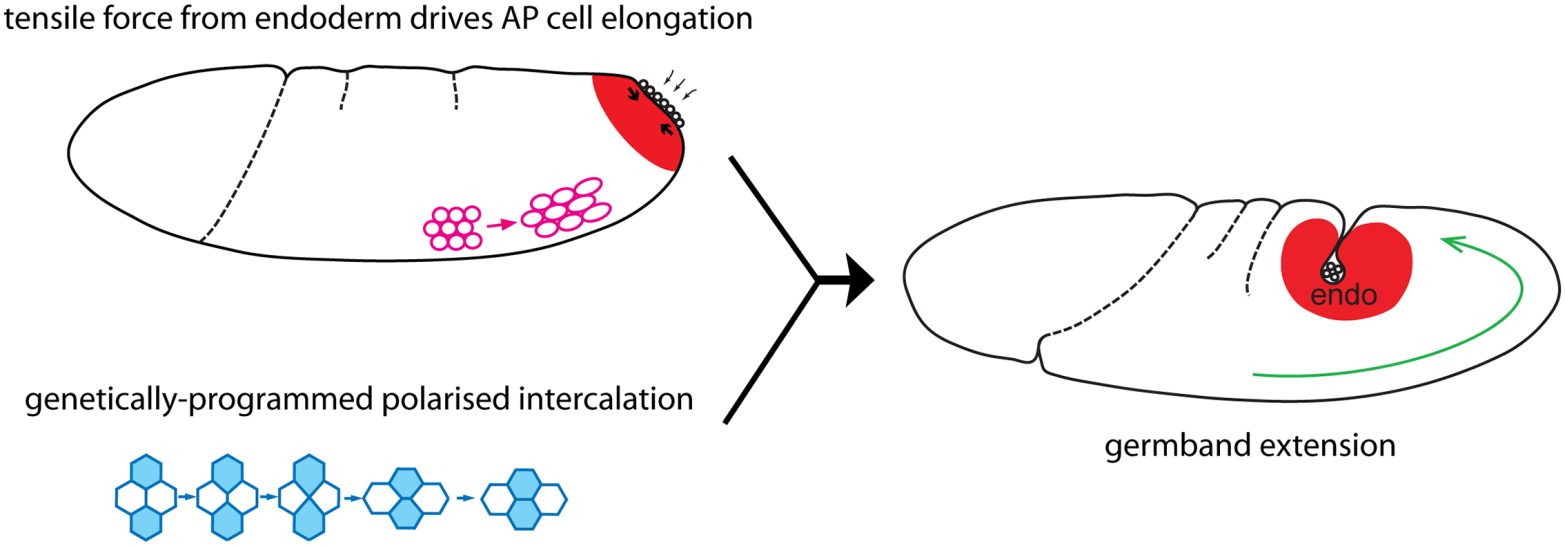
Schematic summary. Our findings indicate that apical constriction and invagination of the endoderm primordium (red region) causes a tensile stress that is propagated to the germband and elongates the cells in AP (pink). Passive AP cell elongation and genetically-programmed polarized cell intercalation (blue) contribute together to *Drosophila* GBE (green arrow). endo, endoderm.

We have investigated the origin of the patterns of planar cell shape changes that we hypothesized previously were the signature of an extrinsic force acting during *Drosophila* axis extension [34]. We showed that the AP-oriented elongation of cell apices contributing to GBE are strongest at the posterior end of the embryo and decrease gradually towards the anterior. AP cell elongation is accompanied by an increase in cell area, suggesting that this gradient of cell shape change arises in response to a planar tensile stress coming from the tail end of the embryo. We found that the patterns of AP cell elongation and cell area increase are eliminated in the absence of posterior endoderm invagination (but not mesoderm invagination), suggesting that this morphogenetic movement is the source of the extrinsic force deforming the germband. We show that in acellular embryos, the cortical Myosin II meshwork flows towards the contracting posterior endoderm region, and that this is accompanied by an increased tension at the posterior. We conclude from these experiments that the apical constriction and invagination of the posterior endoderm primordium generates a tensile stress propagating to the germband and causing the AP cell elongation gradient that contributes to *Drosophila* axis extension (Fig 7).

We can think of two alternative explanations that could challenge this conclusion. First, AP cell elongations could be a secondary consequence of active cell intercalation. However, in AP patterning mutants such as *Kr*, where active polarized cell intercalation is diminished, AP cell elongation is increased rather than decreased [34]. This indicates that active cell intercalation (and AP patterning) is not required for AP cell elongation. Also, cell intercalation rates are high throughout the trunk [34], whereas AP cell elongation is found in a gradient culminating at the posterior (this paper). Therefore, these differing spatial patterns suggest that these two cell behaviours have independent origins. Also in acellular embryos, we observe posteriorward flows of the apical cortex associated with increased tension at the posterior, in absence of polarized cell intercalation. Together, this argues that polarized cell intercalation is not responsible for the gradient of AP cell elongation we observe.

Another possibility is that AP cell elongations are cell-autonomous, that is the result of an active spreading of the germband cells under the control of a genetic program. AP patterning is not required (see above), and the other patterning systems known to operate in the early embryo are the DV and terminal systems [44]. The observed gradient of AP cell elongation is orthogonal to the DV patterning axis and extends outside the terminal domain, so it cannot be explained simply by the activity of either of these systems. We conclude that the most parsimonious explanation is that the AP cell elongation patterns we observe are passive cell behaviours that occur in response to mechanical stresses.

We have found that the AP cell elongation gradient is still present in *twi* mutants in posterior views, refuting our previous model for a role of the mesoderm in producing these cell shape changes, which was based on analyzing anterior views [34]. We think that the source of the discrepancy is that the anterior and posterior views we imaged are further apart in *twi* mutants compared to wild-type, which means that the AP cell elongation gradient was mostly missed in *twi* anterior views. We identify at least one factor, curvature, to explain this difference. The difficulty in registering fields of view between these two genotypes precludes a more detailed comparison of the AP cell elongation gradient. Therefore, we cannot rule out a subtle contribution of mesoderm invagination to GBE. For example, mesoderm invagination, by changing the shape and perhaps the mechanical properties of the germband, might affect how the stress from endoderm invagination propagates throughout the ectoderm. This has some support from the analysis of the AP cell elongation gradient’s slope at specific timepoints, which appear shallower in *twi* mutant (see for example timepoint 7.5 min in Fig 3G”). To be able to compare the gradient of AP cell elongation between the two genotypes, we will need to perform apical cell deformation analysis in whole embryo movies such as the SPIM movies presented in this paper, in order to circumvent the problem of registering fields of view.

Our experiments identify the endoderm primordium as a source of tensile force. Using acellular embryos allowed us to explore how mechanical stresses could be produced by the posterior endoderm. Although they do not have cells, these mutant embryos are able to undergo the initial steps leading to both mesoderm [46] and endoderm invagination (this study). The apical surfaces of the embryo corresponding to the mesoderm and endoderm primordia are seen to enrich Myosin II, contract, and begin to invaginate ([46], this study), as in wild-type embryos [48]. A rigorous quantitative analysis on the mesoderm has demonstrated that the apical forces of constriction are transmitted to the underlying cytoplasm deep in the tissue and are sufficient to promote invagination, showing that cell individualization is dispensable, at least for the initial phases of invagination [46]. Our qualitative study suggests that the forces generated by apical constriction are also transmitted in the plane at the surface of acellular embryos. Using PIV, we visualized surface flows of Myosin II towards the mesoderm and endoderm primordia. Our laser ablation experiments indicate that the flows towards the endoderm primordium are accompanied by an increase in tension at the cortical surface of the acellular embryo. This suggests that apical cell constrictions of the endoderm primordium and the beginning of invagination are able to produce planar forces that pull the adjoining apical surfaces of the germband.

How do stresses transmitted from the apical cortex of constricting endodermal cells translate into a gradient of AP cell elongation in the elongating germband? Epithelial cells of the germband are thought to be connected mechanically to each other through the actomyosin cytoskeleton interacting with components of the apical adherens junctions such as the E-cadherin complexes [29,49]. Thus, tensile stresses caused by apical constriction should propagate through tissues and can conceivably result in mechanically stretching cells over some distance. We find here that germband cells elongate in AP over a distance of at least 150 μm from the site of endoderm constriction (See Fig 1E’). The gradation in AP cell elongation in response to endoderm invagination that we observe might be explained by friction or resistance from the cellular environment. These would prevent forces being instantaneously propagated throughout the germband. Since the germband tissue has to go around the posterior tip of the embryo to elongate, geometry might also have an impact on how forces are transmitted. Finally, we cannot exclude that spatial variation in stiffness of germband cells along the AP axis could cause them to respond differently to mechanical stress.

Endoderm and mesoderm invagination are both triggered by apical constrictions powered by apical networks of actomyosin [48]. We previously detected a stretch of the ectodermal cells in DV behind the invaginating mesoderm [34]. We confirm this in this paper, showing that DV elongation of the germband cells occurs for the first 5–7 min of GBE in wild-type. This is abolished in *twi* mutants in which mesoderm invagination is defective. Thus, germband cells are subjected to two independent tensile forces: one in the DV direction (around the onset of GBE) caused by mesoderm invagination, and another in the AP direction (during early GBE), caused by posterior endoderm invagination. Together, these observations show that the epithelial cells in the germband can respond passively to tensile stress generated in adjacent tissues apically constricting and invaginating, by stretching along the direction of stress.

The directionality of apical cell elongation is strongly constrained to AP for the patterns linked to endoderm invagination and to DV for those linked to mesoderm invagination. Indeed, the patterns of AP cell length change caused by endoderm invagination are not accompanied by much change in DV cell length and vice versa for mesoderm invagination (Compare S2C’ and S2C”Fig). Since both AP and DV cell elongation patterns are accompanied by an increase in cell area (S2A’Fig), this implies that the germband cells must shorten their *z*-axis if they are to maintain a constant cell volume. The maintenance of a constant cell volume throughout gastrulation appears likely, based on recent measurements [37,38]. We cannot access the Z dimension with our analyses of apical cell surface deformation and so verifying that cells do shorten along their *z*-axis will require tracking and analyzing cell shape changes in 3-D.

We had shown previously that the AP cell elongation patterns that we are observing in the germband contribute to axis extension [34]. This was shown by measuring strain rates (deformation) for the whole tissue and decomposing these into the strain rates caused by the cell length change and the strain rates caused by polarized cell intercalation [34,35]. We found that although the predominant behavior extending the germband is polarized cell intercalation, AP cell length changes are contributing significantly (about one-third of the total deformation) early in GBE. A question that remains is why AP cell elongation is temporally limited to early GBE, peaking around 10 min after the onset of GBE (Fig 1F and 1F’). In fact, AP cell elongation ceases rather abruptly at around 15 min after GBE onset (Fig 1E’). SPIM movies indicate that this developmental time (taking into account the difference in temperature for the acquisition of these movies, see Materials and Methods) corresponds to when the posterior midgut invagination becomes cup-shaped and appears to drop down from the surface of the embryo (S3 Movie; see schematics in Fig 4A and Fig 7). A possibility is that force generation from endoderm invagination ceases at this time, perhaps because apical constrictions in the primordium are completed. Alternatively, the presence or not of AP cell elongation in the germband could be a function of the balance between how much the actively elongating germband can push and how much endoderm invagination can pull. In other words, early, the pull from endoderm invagination might be stronger than the push from the extending germband, causing a stress in the germband tissue, which manifests as AP cell elongation. Late, the push from GBE versus the pull from endoderm invagination might be balanced: germband cells would not experience stress anymore and would cease to elongate.

In addition to producing cell shape changes contributing to axis extension, does the endoderm-generated tensile force have other roles in axis extension? The posterior pole of the embryo does not move dorsally in *fog* and *tsl* mutants and is associated with a buckling of the germband [43]. A possible interpretation of this phenotype is that the actively extending germband cannot intrinsically “push” round the corner (or displace presumptive endoderm). So endoderm invagination may have the role of guiding the germband around the posterior tip to overcome the obstacles posed by the surrounding tissues and the embryo geometry. The tensile stress from the endoderm might also facilitate polarized cell intercalation. Whereas DV-shortening of junctions is known to be caused by the intrinsic activity of the actomyosin cytoskeleton, it remains unclear how the AP-oriented nascent junctions elongate at the end of intercalation [9–19,22,26]. A possibility is that the extrinsic tensile force from the endoderm facilitates this AP junctional elongation either by directly exerting tension on the junctions or by indirectly “making space” for cells to intercalate, or in other words by displacing the boundary ahead of the self-deforming tissue [50]. It is also possible that an AP tensile stress could contribute to the nonreversibility of cell intercalation.

Finally, it is remarkable that three morphogenetic movements principally driven by cell-autonomous behaviours, GBE by polarized cell intercalation, and mesoderm and endoderm invagination by apical constriction, are happening so synchronously (Fig 4B). Furthermore, these movements are controlled by three distinct patterning systems: AP, DV, and terminal, respectively, that are understood to function independently of each other at these early stages [44]. It is unclear how the embryo can synchronize these three movements so precisely. One possibility is that there is a yet-undiscovered genetic cross talk between these pathways. However, our findings suggest an alternative explanation, that mechanical coupling between the invaginations of gastrulation and axis extension helps this synchronization. In vertebrate embryos, convergence and extension movements also happen at the same time as other morphogenetic deformations, for example epiboly [3] or neurulation [5], so understanding how morphogenetic movements interact is going to be important to fully understand how embryos are shaped.

## Materials and Methods

### Fly Strains

Transgenic strains were *spider-GFP*, *resille-GFP* [51], *ubi-DE-cad-GFP* [52] and *sqh-GFP*[53]. Mutant alleles were *Kr* [1], *twi* [1], *tsl* [4], the *tsl* deficiency *Df[3R]ED6076* (Flybase and Bloomington Stock Centre), and the acellular mutant characterized in [46].

### Apical Cell Surface Imaging, Tracking, and Deformation Analysis

Anterior movies are taken from [34]. Posterior movies were acquired as follows: late stage five embryos labeled with *ubi-DE-cad-GFP* were imaged ventrally every 30 sec at 20.5 ± 1°C, using a spinning disc confocal. Cell tracking, cell shape, and cell area analyses were performed as before using custom software (oTracks) written in IDL [34,35]. Best-fit ellipses are used to represent cell shapes and to calculate cell deformation. For statistics, we used a mixed-effects model as before [34].

### Whole Embryo Imaging

Late stage five embryos labeled with *spider-GFP* and/or *resille-GFP* were mounted in 1.5% low melting point agarose and imaged using mSPIM [39]. Embryos were rotated to image four perpendicular views, which were reconstructed into a whole embryo image stack post-acquisition [54]. Image stacks were acquired every 30 sec at 28–30°C for 60 min. Reconstructed movies of three wild-type and three *twi* mutants were viewed in 4-D in custom software (Browser and Tracer) written in Interactive Data Language (IDL, Exelis) [55] to map timings of morphogenetic movements. Scatter graphs were plotted in Prism (GraphPad).

### PIV and Laser Ablation in Acellular Embryos

PIV was performed to visualize Myosin II flows at the embryo scale and also at a smaller scale to analyze relaxation of the tissue after laser ablation in acellular embryos.

Further details on the Materials and Methods are in S1 Text.

## Supporting Information

S1 Data Excel file for graphs in Fig 1 and S1 Fig. Data for each graph is given in a separate sheet containing raw data values of *x*- and *y*-axes, along with the corresponding cell and embryo identifier for each data point when appropriate (cell identifier and embryo name, respectively). Each sheet is labelled with the relevant figure panel number.
(XLSX)

S2 Data Excel file for graphs in Fig 2 and S2 Fig. Data for each graph is given in a separate sheet containing raw data values of *x*- and *y*-axes, along with the corresponding cell and embryo identifier for each data point when appropriate (cell identifier and embryo name, respectively). Each sheet is labelled with the relevant figure panel number and, when a graph depicts more than one data set (e.g., different timepoints), the data set label.
(XLSX)

S3 Data Excel file for graphs in Fig 3 and S3 Fig. Data for each graph is given in a separate sheet containing raw data values of *x*- and *y*-axes, along with the corresponding cell and embryo identifier for each data point when appropriate (cell identifier and embryo name, respectively). Each sheet is labelled with the relevant figure panel number and, when a graph depicts more than one data set (e.g., different genotypes), the data set label.
(XLSX)

S4 Data Excel file for graph in Fig 4B. Times of GBE are shown in minutes for each morphogenetic event (rows) and embryo (columns).
(XLSX)

S5 Data Excel file for graphs in Fig 5 and S5 Fig. Data for each graph is given in a separate sheet containing raw data values of *x* and *y*-axes, along with the corresponding cell and embryo identifier for each data point when appropriate (cell identifier and embryo name, respectively). Each sheet is labelled with the relevant figure panel number and, when a graph depicts more than one data set (e.g., different genotypes), the data set label.
(XLSX)

S6 Data Excel file for graph in Fig 6E. Normalized relaxation speeds (μm/s) are given for each data point for each ablation class.
(XLSX)

S1 Fig AP cell length change in individual movies of wild-type embryos for anterior and posterior views and cell tracking information. (A, B) Spatiotemporal maps summarizing AP cell length change over the first 30 min of GBE (*y*-axis), and as a function of cell position in the AP axis (*x*-axis), for anterior and posterior views, for each movie collected. The position in the AP axis is measured from the anterior and posterior landmarks defined in Fig 1. Note that the cells analyzed for the anterior field of views do not include those deformed by the cephalic furrow (See Fig 1C and 1D). (C) Graph showing the total number of cells analyzed per timepoint (*y*-axis), as a function of time after GBE onset (*x*-axis), for all five movies of anterior views. (C’) Example movie frame from *wtLB009* showing how long a given cell has been tracked at timepoint 30 min after GBE onset. The cells that have been tracked longest (30 min) are shown in red in the heat scale, while the cells that have just started to be tracked are shown in blue. (D, D’) Same as C, C’, but for posterior views. For D, the number of movies is four, and for D’, the example movie frame corresponds to *wtCL051010*. Scale bars are 50 microns. Data associated with this Fig can be found in S1 Data.
(TIF)

S2 Fig Cell area change and comparison of AP and DV cell length change in wild-type embryos for anterior and posterior views. (A, A’) Spatiotemporal maps summarizing cell area change as a function of time after GBE onset (*y*-axis) and position in the AP axis, for anterior (A) and posterior views (A’), averaged for five and four wild-type embryos, respectively. The increase of cell area from 0 to 5–7 min is caused by the germband cells stretching in DV behind the invaginating mesoderm (absent in *twi* maps, see S3C and S3C’ Fig). (B–C”) Comparison of AP and DV cell length change for anterior (B–B”) and posterior views (C–C”). (B, C) Graphs summarizing AP (blue) and DV (red) cell length change as a function of time after GBE onset (*x*-axis), for anterior and posterior views. (B’, C’) Spatiotemporal maps summarizing AP cell length change as a function of time after GBE onset (*y*-axis) and position in the AP axis, for anterior and posterior views. (B”, C”) Corresponding maps for DV cell length change. The signal from 0 to 5–7 min is caused by the germband cells stretching in DV behind the invaginating mesoderm (absent in *twi* maps, see S3D” and S3E” Fig). Data associated with this Fig can be found in S2 Data.
(TIF)

S3 Fig Cell area change and comparison of AP and DV cell length change in *twi* mutant embryos for anterior and posterior views. (A, B) Spatiotemporal maps summarizing AP cell length change over the first 30 min of GBE (*y*-axis) and as a function of cell position in the AP axis (*x*-axis) for *twi* mutant embryos, for anterior (A) and posterior views (B), for each movie collected per genotype. The position in the AP axis is measured from the anterior and posterior landmarks defined in Fig 1. Note that the cells analyzed for the anterior field of views do not include those deformed by the cephalic furrow (See wild-type example in Fig 1C and 1D). (C, C’) Spatiotemporal maps summarizing cell area change as a function of time after GBE onset (*y*-axis) and position in the AP axis, for anterior (C) and posterior views (C’), averaged for five and three *twi* mutant embryos, respectively. (D–E”) Comparison of AP and DV cell length change for anterior (D–D”) and posterior views (E–E”) for *twi* mutant embryos. (D, E) Graphs summarizing AP (blue) and DV (red) cell length change as a function of time after GBE onset (*x*-axis), for anterior and posterior views. (D’, E’) Spatiotemporal maps summarizing AP cell length change as a function of time after GBE onset (*y*-axis) and position in the AP axis, for anterior and posterior views. (D”, E”) Corresponding maps for DV cell length change. Data associated with this Figure can be found in S3 Data.
(TIF)

S4 Fig Embryo shapes comparisons between anterior views of wild-type and *twi* mutant embryos. (A, B) Movie frames at timepoint 10 min after GBE onset for the anterior views, for wild-type (A) and *twi* mutant embryos (B). (C, C’) Drawn outlines of the five wild-type and five *twi* mutant embryos: the curvatures in *twi* embryos are less pronounced and the embryos wider compared to wild type.
(TIF)

S5 Fig Ectopic folds during axis extension in *fog* mutant embryos and individual movies for *Kr* and *Kr; tsl* mutants. (A) Frames from a movie of a *fog* mutant embryo, at 5, 10, and 20 min after GBE onset. Folds start forming at ectopic sites early in axis extension. In this example, two deep folds form on one side of the embryo (arrows). (B) Corresponding spatiotemporal map summarizing AP cell length change over the first 20 mins of GBE (*y*-axis) and as a function of cell position in the AP axis (*x*-axis). The two folds seen in the movie are detected as AP cell length change on either side of the folds (indicated by arrows), from about 7 min onwards. There is no data available at the position of the folds, because the cells cannot be tracked. Note that outside the fold-induced signal, there is no obvious AP cell elongation gradient detectable in this mutant embryo. (C, E) Spatiotemporal maps summarizing AP cell length change over the first 30 mins of GBE (*y*-axis) and as a function of cell position in the AP axis (*x*-axis), for *Kr* (C) and *Kr; tsl* mutants (E), for each of the three movies collected per genotype. (D, F) Corresponding spatiotemporal maps summarizing cell area changes for each genotypes.
(TIF)

S6 Fig Myosin II and E-cadherin localization in acellular embryos. Scale bars are 20 microns for all panels. (A–D’) Posterior lateral views of fixed acellular embryos stained against E-cadherin and Myosin II (using antibody against mono-phosphorylated MRLC). Two stages are shown, just before gastrulation movements start (estimated stage five; A, A’, C, C’) and during gastrulation (estimated stage seven; B, B’, D, D’). For each stage, a projection of confocal sections shows the signal close to the surface (0–2 μm, A–B’) and a little deeper (> 3 μm, C–D’). The confocal sections used for each projection are shown by a red bracket in the reconstructed cross-section underneath each panel. The position of the cross-sections is indicated by a red line in each panel. PC are indicated. (E) Example of a laser ablation experiment for a DV-oriented cut at the posterior of an acellular embryo. Confocal images of the Myosin II signal are collected every 0.742 ms (timepoints displayed are indicated on panels) for 20 frames before and 120 frames after the cut (time zero, no image is acquired during ablation). Note that the images shown here are destriped and denoised (see supplementary Materials and Methods). The cut is seen as a gap in the Myosin II meshwork. The timepoints just before and after the cut are overlaid to show the displacement of the signal (merge). The gap caused by ablation continues to open for approximately 10–15 sec. Later on, new Myosin II signal moves in, eventually “repairing” the gap by about 1 min post-ablation. (F–G’) PIV analysis of Myosin II flows for the ablation experiment shown in E, overlayed on Myosin II signal (the images shown here are destriped but not denoised). The optical flows are represented with green arrows, which show displacement between the timepoint shown and the previous one, scaled by a factor of 25 (only 1 arrow in every 3 x 3 grid is visualized). Only the flow component perpendicular to the cut are visualized and analyzed. The velocity of optical flows are analyzed within a small region around each cut (white polygon), and shown for the –0.74 s timepoint before the cut (F, and close-up in F’) and the 1.5 s timepoint after the cut (G, and close-up in G’). Magenta arrows represent the average velocity of the optical flows for each side of the ablation line (dashed) for this region. The average velocities before the cut are subtracted from those after the cut to correct for translation (see supplementary Materials and Methods) giving the normalized relaxation speed shown in Fig 6E.
(TIF)

S1 Movie Example posterior view wild-type movie (wtCL051010) (left panel) and corresponding cell shape strain rates on tracked cells (right panel). Data is shown from 0 to 30 min of GBE. Left panel: Projected confocal z-stack of a wild-type embryo labelled with *ubi-DE-cad-GFP*. Right panel: Tracked cell outlines (grey), after exclusion of unwanted mesodermal and mesectodermal cells, with color-coded scale of blue to red, 0–0.06 pp/min, illustrating AP cell shape change of each analyzed cell at that time point.
(MOV)

S2 Movie Example posterior view *twi* movie (twiCL140411) (left panel) and corresponding cell shape strain rates on tracked cells (right panel). Data is shown from 0 to 30 min of GBE. Left panel: Projected confocal z-stack of a *twi* embryo labelled with *ubi-DE-cad-GFP*. Right panel: Tracked cell outlines (grey), after exclusion of unwanted mesodermal and mesectodermal cells, with color-coded scale of blue to red, 0–0.06 pp/min, illustrating AP cell shape change of each analyzed cell at that time point.
(MOV)

S3 Movie Example lateral view from mSPIM movie illustrating the temporal mapping of GBE (yellow), endoderm invagination (red), dorsal fold formation (green) and dorsal contraction (magenta). Lateral slice of reconstructed mSPIM movie of a wild-type embryo labeled with the whole-membrane markers *resille-GFP* and *spider-GFP* from 10 min before, to 10 min after the start of GBE. Anterior end is to the left, ventral side is to the bottom of the movie. Mapping of AP movement of individual ventral cells situated at the anterior and posterior of the germband (yellow dots), the two yellow cells initially move towards and then away from each other, the latter indicating the onset of GBE; mapping of apical constriction of two groups of cells at the posterior end of the embryo either side of the PC (red dots) indicating the onset of endoderm invagination; mapping of the apical surface of a dorsal cell in comparison to its initial position (green dots) on the dorsal surface indicating the initiation of the posterior dorsal fold; mapping of the movement of individual dorsal cells situated at the anterior and posterior of the dorsal side (magenta dots), cells get closer together indicating a dorsal contraction. See also Fig 4.
(MOV)

S4 Movie Example ventral view from mSPIM movie illustrating the temporal mapping of mesoderm sealing. Ventral slice of reconstructed mSPIM movie of a wild-type embryo labeled with the whole-membrane markers *resille-GFP* and *spider-GFP* from 10 minutes before, to 10 min after the start of GBE. The ventral furrow can be seen during the process of apical constriction and subsequent mesoderm sealing. In this example mesoderm sealing starts towards the anterior (left) and quickly propagates towards the posterior (right). See also Fig 4.
(MOV)

S5 Movie Example posterior view *Kr* movie (KrCL051112) (left panel) and corresponding cell shape strain rates on tracked cells (right panel). Data is shown from 0 to 30 min of GBE. Left panel: Projected confocal z-stack of a *Kr* embryo labelled with *ubi-DE-cad-GFP*. Right panel: Tracked cell outlines (grey), after exclusion of unwanted mesodermal and mesectodermal cells, with color-coded scale of blue to red, 0–0.06 pp/min, illustrating AP cell shape change of each analyzed cell at that time point.
(MOV)

S6 Movie Example posterior view *Kr; tsl* movie (KrtslCL040713) (left panel) and corresponding cell shape strain rates on tracked cells (right panel). Data is shown from 0 to 30 min of GBE. Left panel: Projected confocal z-stack of a *Kr; tsl* embryo labelled with *ubi-DE-cad-GFP*. Right panel: Tracked cell outlines (grey), after exclusion of unwanted mesodermal and mesectodermal cells, with color-coded scale of blue to red, 0–0.06 pp/min, illustrating AP cell shape change of each analyzed cell at that time point.
(AVI)

S7 Movie Anterior view *fog* movie. Projected confocal z-stack of a *fog* embryo labelled with *ubi-DE-cad-GFP* illustrating formation of folds during GBE in this mutant. Data is shown from 0 to 30 min of GBE.
(MOV)

S8 Movie Acellular embryo movie and PIV analysis of flows. Data is shown from 0 to 32 min from the start of gastrulation movements. Top panel: Z-projected confocal stack of an acellular embryo expressing *sqh-GFP* to label Myosin II. Bottom panel: same embryo as in top panel, with visualization of Myosin II flows by PIV overlayed.
(AVI)

S1 Text Extended Materials and Methods. (DOCX)

## Abbreviations

AP: anteroposterior
DV: dorsoventral
*fog*: *folded gastrulation*
GBE: germband extension
*Kr*: *Kruppel*
PC: pole cells
PIV: particle imaging velocimetry
SPIM: selective plane illumination microscopy
mSPIM: multidirectional SPIM
*tsl*: *torso-like*
*twist*: *twi*

## References

[1] Keller R. Shaping the vertebrate body plan by polarized embryonic cell movements. Science. 2002;298(5600):1950–4. Epub 2002/12/10. 12471247 10.1126/science.1079478

[2] Tada M, Heisenberg CP. Convergent extension: using collective cell migration and cell intercalation to shape embryos. Development. 2012;139(21):3897–904. Epub 2012/10/11. 10.1242/dev.073007 23048180

[3] Solnica-Krezel L, Sepich DS. Gastrulation: making and shaping germ layers. Annual review of cell and developmental biology. 2012;28:687–717. Epub 2012/07/19. 10.1146/annurev-cellbio-092910-154043 22804578

[4] Glickman NS, Kimmel CB, Jones MA, Adams RJ. Shaping the zebrafish notochord. Development. 2003;130(5):873–87. Epub 2003/01/23. 12538515 10.1242/dev.00314

[5] Copp AJ, Stanier P, Greene ND. Neural tube defects: recent advances, unsolved questions, and controversies. Lancet neurology. 2013;12(8):799–810. Epub 2013/06/25. 10.1016/S1474-4422(13)70110-8 23790957 PMC4023229

[6] Wang J, Mark S, Zhang X, Qian D, Yoo SJ, Radde-Gallwitz K, et al Regulation of polarized extension and planar cell polarity in the cochlea by the vertebrate PCP pathway. Nature genetics. 2005;37(9):980–5. Epub 2005/08/24. 16116426 10.1038/ng1622 PMC1413588

[7] Lienkamp SS, Liu K, Karner CM, Carroll TJ, Ronneberger O, Wallingford JB, et al Vertebrate kidney tubules elongate using a planar cell polarity-dependent, rosette-based mechanism of convergent extension. Nature genetics. 2012;44(12):1382–7. Epub 2012/11/13. 10.1038/ng.2452 23143599 PMC4167614

[8] Wallingford JB. Planar cell polarity and the developmental control of cell behavior in vertebrate embryos. Annual review of cell and developmental biology. 2012;28:627–53. Epub 2012/08/22. 10.1146/annurev-cellbio-092910-154208 22905955

[9] Zallen JA, Wieschaus E. Patterned gene expression directs bipolar planar polarity in Drosophila. Developmental cell. 2004;6(3):343–55. Epub 2004/03/20. 15030758 10.1016/s1534-5807(04)00060-7

[10] Bertet C, Sulak L, Lecuit T. Myosin-dependent junction remodelling controls planar cell intercalation and axis elongation. Nature. 2004;429(6992):667–71. Epub 2004/06/11. 15190355 10.1038/nature02590

[11] Blankenship JT, Backovic ST, Sanny JS, Weitz O, Zallen JA. Multicellular rosette formation links planar cell polarity to tissue morphogenesis. Developmental cell. 2006;11(4):459–70. Epub 2006/10/03. 17011486 10.1016/j.devcel.2006.09.007

[12] Fernandez-Gonzalez R, Simoes Sde M, Roper JC, Eaton S, Zallen JA. Myosin II dynamics are regulated by tension in intercalating cells. Developmental cell. 2009;17(5):736–43. Epub 2009/11/03. 10.1016/j.devcel.2009.09.003 19879198 PMC2854079

[13] Simoes Sde M, Blankenship JT, Weitz O, Farrell DL, Tamada M, Fernandez-Gonzalez R, et al Rho-kinase directs Bazooka/Par-3 planar polarity during Drosophila axis elongation. Developmental cell. 2010;19(3):377–88. Epub 2010/09/14. 10.1016/j.devcel.2010.08.011 20833361 PMC3131216

[14] Tamada M, Farrell DL, Zallen JA. Abl regulates planar polarized junctional dynamics through beta-catenin tyrosine phosphorylation. Developmental cell. 2012;22(2):309–19. Epub 2012/02/22. 10.1016/j.devcel.2011.12.025 22340496 PMC3327890

[15] Simoes Sde M, Mainieri A, Zallen JA. Rho GTPase and Shroom direct planar polarized actomyosin contractility during convergent extension. The Journal of cell biology. 2014;204(4):575–89. Epub 2014/02/19. 10.1083/jcb.201307070 24535826 PMC3926966

[16] Rauzi M, Verant P, Lecuit T, Lenne PF. Nature and anisotropy of cortical forces orienting Drosophila tissue morphogenesis. Nature cell biology. 2008;10(12):1401–10. Epub 2008/11/04. 10.1038/ncb1798 18978783

[17] Rauzi M, Lenne PF, Lecuit T. Planar polarized actomyosin contractile flows control epithelial junction remodelling. Nature. 2010;468(7327):1110–4. Epub 2010/11/12. 10.1038/nature09566 21068726

[18] Levayer R, Pelissier-Monier A, Lecuit T. Spatial regulation of Dia and Myosin-II by RhoGEF2 controls initiation of E-cadherin endocytosis during epithelial morphogenesis. Nature cell biology. 2011;13(5):529–40. Epub 2011/04/26. 10.1038/ncb2224 21516109

[19] Levayer R, Lecuit T. Oscillation and polarity of E-cadherin asymmetries control actomyosin flow patterns during morphogenesis. Developmental cell. 2013;26(2):162–75. Epub 2013/07/23. 10.1016/j.devcel.2013.06.020 23871590

[20] Warrington SJ, Strutt H, Strutt D. The Frizzled-dependent planar polarity pathway locally promotes E-cadherin turnover via recruitment of RhoGEF2. Development. 2013;140(5):1045–54. Epub 2013/02/01. 10.1242/dev.088724 23364328 PMC3583042

[21] Irvine KD, Wieschaus E. Cell intercalation during Drosophila germband extension and its regulation by pair-rule segmentation genes. Development. 1994;120(4):827–41. Epub 1994/04/01. 7600960 10.1242/dev.120.4.827

[22] Pare AC, Vichas A, Fincher CT, Mirman Z, Farrell DL, Mainieri A, et al A positional Toll receptor code directs convergent extension in Drosophila. Nature. 2014;515(7528):523–7. Epub 2014/11/05. 10.1038/nature13953 25363762 PMC4943584

[23] Shindo A, Wallingford JB. PCP and septins compartmentalize cortical actomyosin to direct collective cell movement. Science. 2014;343(6171):649–52. Epub 2014/02/08. 10.1126/science.1243126 24503851 PMC4167615

[24] Nishimura T, Honda H, Takeichi M. Planar cell polarity links axes of spatial dynamics in neural-tube closure. Cell. 2012;149(5):1084–97. Epub 2012/05/29. 10.1016/j.cell.2012.04.021 22632972

[25] Rozbicki E, Chuai M, Karjalainen AI, Song F, Sang HM, Martin R, et al Myosin-II-mediated cell shape changes and cell intercalation contribute to primitive streak formation. Nature cell biology. 2015;17(4):397–408. Epub 2015/03/31. 10.1038/ncb3138 25812521 PMC4886837

[26] Tepass U. Developmental biology: Polarize to elongate. Nature. 2014;515(7528):499–501. Epub 2014/11/05. 10.1038/nature13937 25363775

[27] Zhang H, Labouesse M. Signalling through mechanical inputs: a coordinated process. Journal of cell science. 2012;125(Pt 13):3039–49. Epub 2012/08/30. 10.1242/jcs.093666 22929901

[28] Heisenberg CP, Bellaiche Y. Forces in tissue morphogenesis and patterning. Cell. 2013;153(5):948–62. Epub 2013/05/28. 10.1016/j.cell.2013.05.008 23706734

[29] Lye CM, Sanson B. Tension and epithelial morphogenesis in Drosophila early embryos. Current topics in developmental biology. 2011;95:145–87. Epub 2011/04/20. 10.1016/B978-0-12-385065-2.00005-0 21501751

[30] Zhang H, Landmann F, Zahreddine H, Rodriguez D, Koch M, Labouesse M. A tension-induced mechanotransduction pathway promotes epithelial morphogenesis. Nature. 2011;471(7336):99–103. Epub 2011/03/04. 10.1038/nature09765 21368832

[31] Haigo SL, Bilder D. Global tissue revolutions in a morphogenetic movement controlling elongation. Science. 2011;331(6020):1071–4. Epub 2011/01/08. 10.1126/science.1199424 21212324 PMC3153412

[32] Aigouy B, Farhadifar R, Staple DB, Sagner A, Roper JC, Julicher F, et al Cell flow reorients the axis of planar polarity in the wing epithelium of Drosophila. Cell. 2010;142(5):773–86. Epub 2010/09/04. 10.1016/j.cell.2010.07.042 20813263

[33] Sugimura K, Ishihara S. The mechanical anisotropy in a tissue promotes ordering in hexagonal cell packing. Development. 2013;140(19):4091–101. Epub 2013/09/21. 10.1242/dev.094060 24046322

[34] Butler LC, Blanchard GB, Kabla AJ, Lawrence NJ, Welchman DP, Mahadevan L, et al Cell shape changes indicate a role for extrinsic tensile forces in Drosophila germ-band extension. Nature cell biology. 2009;11(7):859–64. Epub 2009/06/09. 10.1038/ncb1894 19503074 PMC7617986

[35] Blanchard GB, Kabla AJ, Schultz NL, Butler LC, Sanson B, Gorfinkiel N, et al Tissue tectonics: morphogenetic strain rates, cell shape change and intercalation. Nature methods. 2009;6(6):458–64. Epub 2009/05/05. 10.1038/nmeth.1327 19412170 PMC4894466

[36] dos Santos G, Schroeder AJ, Goodman JL, Strelets VB, Crosby MA, Thurmond J, et al FlyBase: introduction of the Drosophila melanogaster Release 6 reference genome assembly and large-scale migration of genome annotations. Nucleic acids research. 2015;43(Database issue):D690–7. Epub 2014/11/16. 10.1093/nar/gku1099 25398896 PMC4383921

[37] Khan Z, Wang YC, Wieschaus EF, Kaschube M. Quantitative 4D analyses of epithelial folding during Drosophila gastrulation. Development. 2014;141(14):2895–900. Epub 2014/06/21. 10.1242/dev.107730 24948599 PMC4197613

[38] Gelbart MA, He B, Martin AC, Thiberge SY, Wieschaus EF, Kaschube M. Volume conservation principle involved in cell lengthening and nucleus movement during tissue morphogenesis. Proceedings of the National Academy of Sciences of the United States of America. 2012;109(47):19298–303. Epub 2012/11/09. 10.1073/pnas.1205258109 23134725 PMC3511084

[39] Huisken J, Stainier DY. Selective plane illumination microscopy techniques in developmental biology. Development. 2009;136(12):1963–75. Epub 2009/05/26. 10.1242/dev.022426 19465594 PMC2685720

[40] Foe VE. Mitotic domains reveal early commitment of cells in Drosophila embryos. Development. 1989;107(1):1–22. Epub 1989/09/01. 2516798

[41] Wang YC, Khan Z, Kaschube M, Wieschaus EF. Differential positioning of adherens junctions is associated with initiation of epithelial folding. Nature. 2012;484(7394):390–3. Epub 2012/03/30. 10.1038/nature10938 22456706 PMC3597240

[42] Pope KL, Harris TJ. Control of cell flattening and junctional remodeling during squamous epithelial morphogenesis in Drosophila. Development. 2008;135(13):2227–38. Epub 2008/05/30. 10.1242/dev.019802 18508861

[43] Sweeton D, Parks S, Costa M, Wieschaus E. Gastrulation in Drosophila: the formation of the ventral furrow and posterior midgut invaginations. Development. 1991;112(3):775–89. Epub 1991/07/01. 1935689 10.1242/dev.112.3.775

[44] St Johnston D, Nusslein-Volhard C. The origin of pattern and polarity in the Drosophila embryo. Cell. 1992;68(2):201–19. Epub 1992/01/24. 1733499 10.1016/0092-8674(92)90466-p

[45] Rice TB, Garen A. Localized defects of blastoderm formation in maternal effect mutants of Drosophila. Developmental biology. 1975;43(2):277–86. Epub 1975/04/01. 805069 10.1016/0012-1606(75)90027-5

[46] He B, Doubrovinski K, Polyakov O, Wieschaus E. Apical constriction drives tissue-scale hydrodynamic flow to mediate cell elongation. Nature. 2014;508(7496):392–6. Epub 2014/03/05. 10.1038/nature13070 24590071 PMC4111109

[47] Acharya S, Laupsien P, Wenzl C, Yan S, Grosshans J. Function and dynamics of slam in furrow formation in early Drosophila embryo. Developmental biology. 2014;386(2):371–84. Epub 2013/12/26. 10.1016/j.ydbio.2013.12.022 24368071

[48] Martin AC, Goldstein B. Apical constriction: themes and variations on a cellular mechanism driving morphogenesis. Development. 2014;141(10):1987–98. Epub 2014/05/08. 10.1242/dev.102228 24803648 PMC4011084

[49] Martin AC, Gelbart M, Fernandez-Gonzalez R, Kaschube M, Wieschaus EF. Integration of contractile forces during tissue invagination. The Journal of cell biology. 2010;188(5):735–49. Epub 2010/03/03. 10.1083/jcb.200910099 20194639 PMC2835944

[50] Davidson LA, Joshi SD, Kim HY, von Dassow M, Zhang L, Zhou J. Emergent morphogenesis: elastic mechanics of a self-deforming tissue. Journal of biomechanics. 2010;43(1):63–70. Epub 2009/10/10. 10.1016/j.jbiomech.2009.09.010 19815213 PMC2813421

[51] Morin X, Daneman R, Zavortink M, Chia W. A protein trap strategy to detect GFP-tagged proteins expressed from their endogenous loci in Drosophila. Proceedings of the National Academy of Sciences of the United States of America. 2001;98(26):15050–5. Epub 2001/12/14. 11742088 10.1073/pnas.261408198 PMC64981

[52] Oda H, Tsukita S. Real-time imaging of cell-cell adherens junctions reveals that Drosophila mesoderm invagination begins with two phases of apical constriction of cells. Journal of cell science. 2001;114(Pt 3):493–501. Epub 2001/02/15. 11171319 10.1242/jcs.114.3.493

[53] Jordan P, Karess R. Myosin light chain-activating phosphorylation sites are required for oogenesis in Drosophila. The Journal of cell biology. 1997;139(7):1805–19. Epub 1998/02/07. 9412474 10.1083/jcb.139.7.1805 PMC2132636

[54] Preibisch S, Saalfeld S, Schindelin J, Tomancak P. Software for bead-based registration of selective plane illumination microscopy data. Nature methods. 2010;7(6):418–9. Epub 2010/05/29. 10.1038/nmeth0610-418 20508634

[55] England SJ, Blanchard GB, Mahadevan L, Adams RJ. A dynamic fate map of the forebrain shows how vertebrate eyes form and explains two causes of cyclopia. Development. 2006;133(23):4613–7. Epub 2006/11/03. 17079266 10.1242/dev.02678

