# Supplementary figures and images for: Mechanical Coupling between Endoderm Invagination and Axis Extension in Drosophila
Source: PLoS Biol. 2015 Nov 6;13(11):e1002292. doi: 10.1371/journal.pbio.1002292 (PMC4636290; doi:10.1371/journal.pbio.1002292)

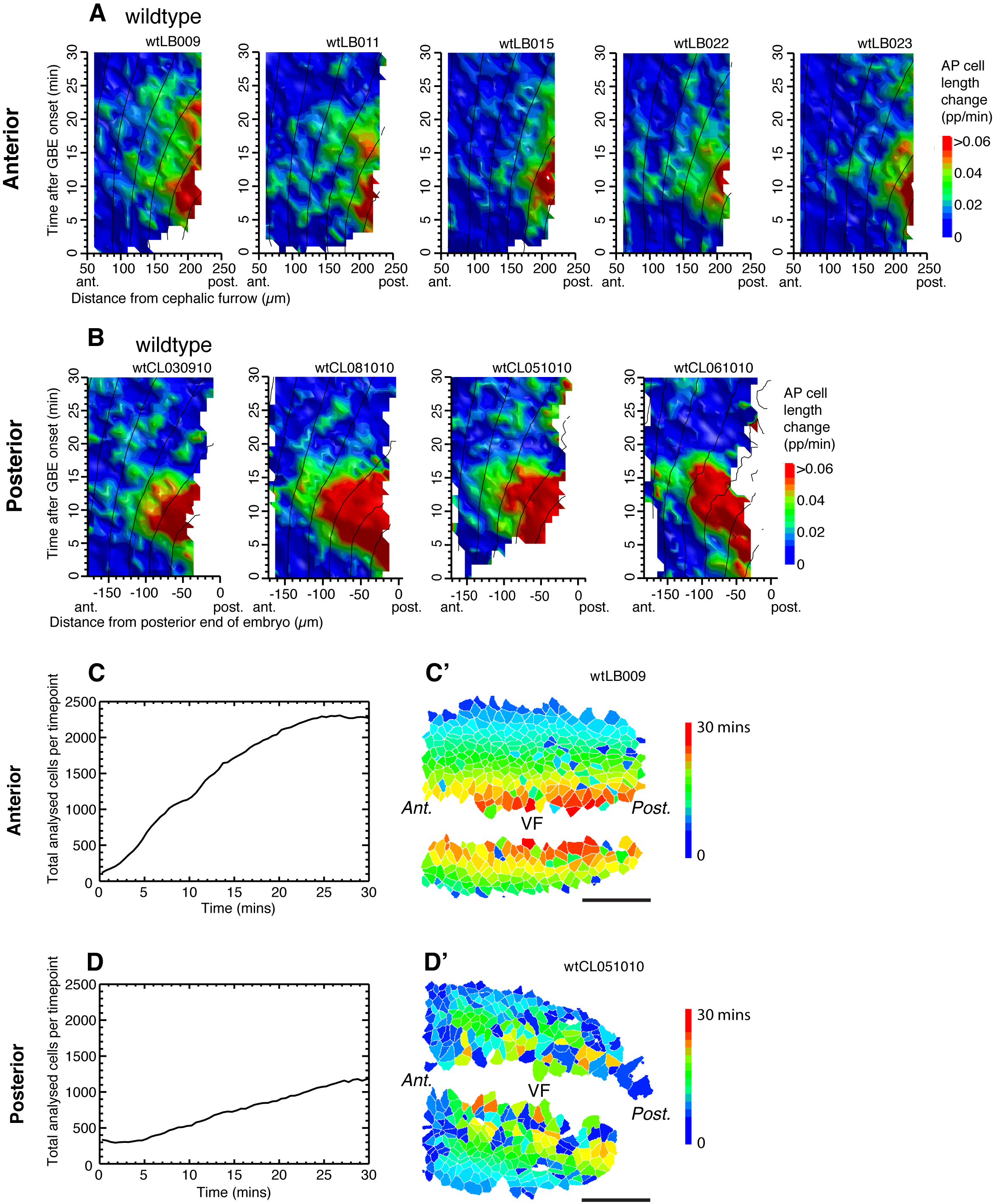

Supplement: S1 Fig — (A, B) Spatiotemporal maps summarizing AP cell length change over the first 30 min of GBE (y-axis), and as a function of cell position in the AP axis (x-axis), for anterior and posterior views, for each movie collected. The position in the AP axis is measured from the anterior and posterior landmarks defined in Fig 1. Note that the cells analyzed for the anterior field of views do not include those deformed by the cephalic furrow (See Fig 1C and 1D). (C) Graph showing the total number of cells analyzed per timepoint (y-axis), as a function of time after GBE onset (x-axis), for all five movies of anterior views. (C’) Example movie frame from wtLB009 showing how long a given cell has been tracked at timepoint 30 min after GBE onset. The cells that have been tracked longest (30 min) are shown in red in the heat scale, while the cells that have just started to be tracked are shown in blue. (D, D’) Same as C, C’, but for posterior views. For D, the number of movies is four, and for D’, the example movie frame corresponds to wtCL051010. Scale bars are 50 microns. Data associated with this Fig can be found in S1 Data. (TIF) [file pbio.1002292.s007.tif]

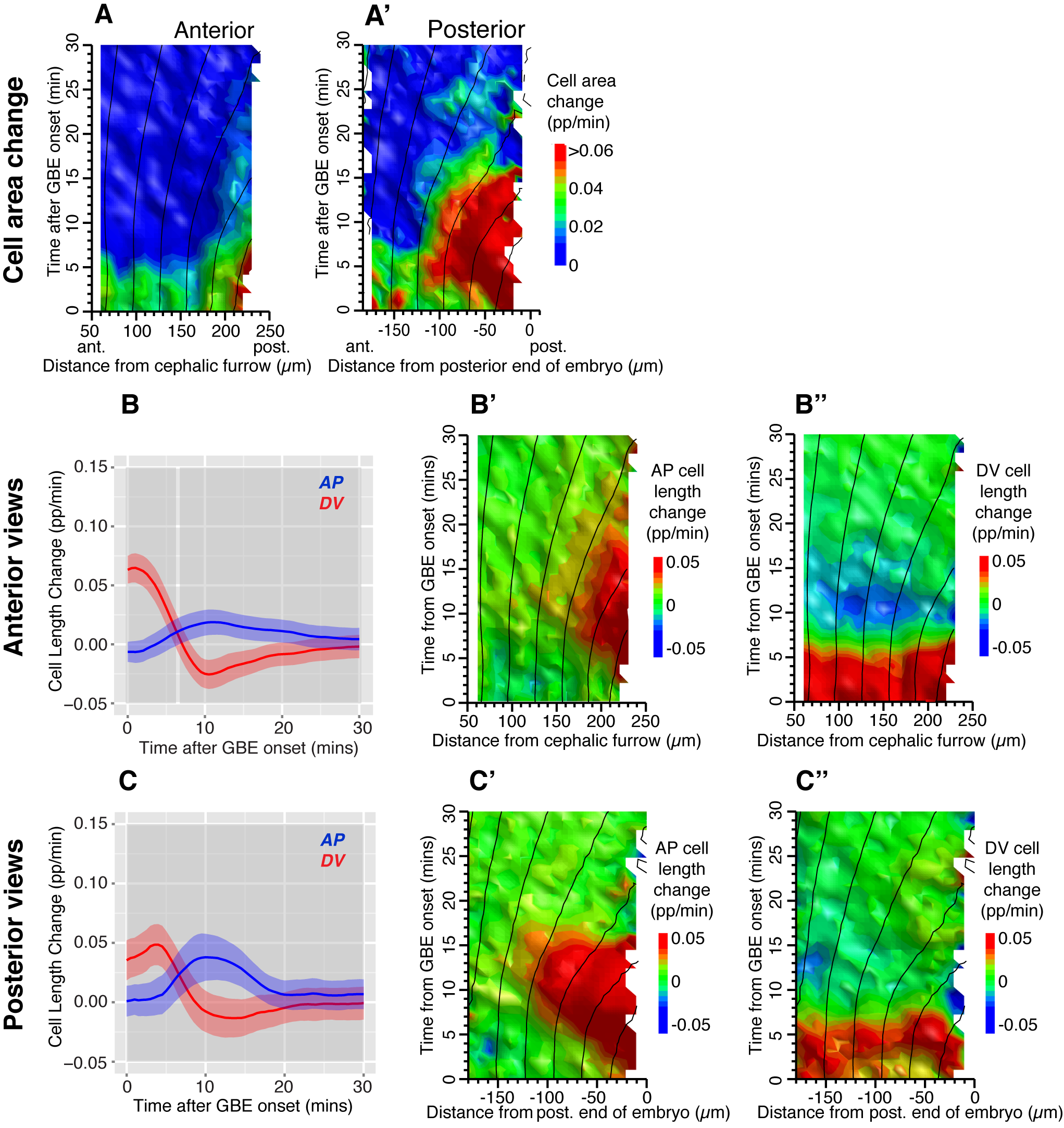

Supplement: S2 Fig — (A, A’) Spatiotemporal maps summarizing cell area change as a function of time after GBE onset (y-axis) and position in the AP axis, for anterior (A) and posterior views (A’), averaged for five and four wild-type embryos, respectively. The increase of cell area from 0 to 5–7 min is caused by the germband cells stretching in DV behind the invaginating mesoderm (absent in twi maps, see S3C and S3C’ Fig). (B–C”) Comparison of AP and DV cell length change for anterior (B–B”) and posterior views (C–C”). (B, C) Graphs summarizing AP (blue) and DV (red) cell length change as a function of time after GBE onset (x-axis), for anterior and posterior views. (B’, C’) Spatiotemporal maps summarizing AP cell length change as a function of time after GBE onset (y-axis) and position in the AP axis, for anterior and posterior views. (B”, C”) Corresponding maps for DV cell length change. The signal from 0 to 5–7 min is caused by the germband cells stretching in DV behind the invaginating mesoderm (absent in twi maps, see S3D” and S3E” Fig). Data associated with this Fig can be found in S2 Data. (TIF) [file pbio.1002292.s008.tif]

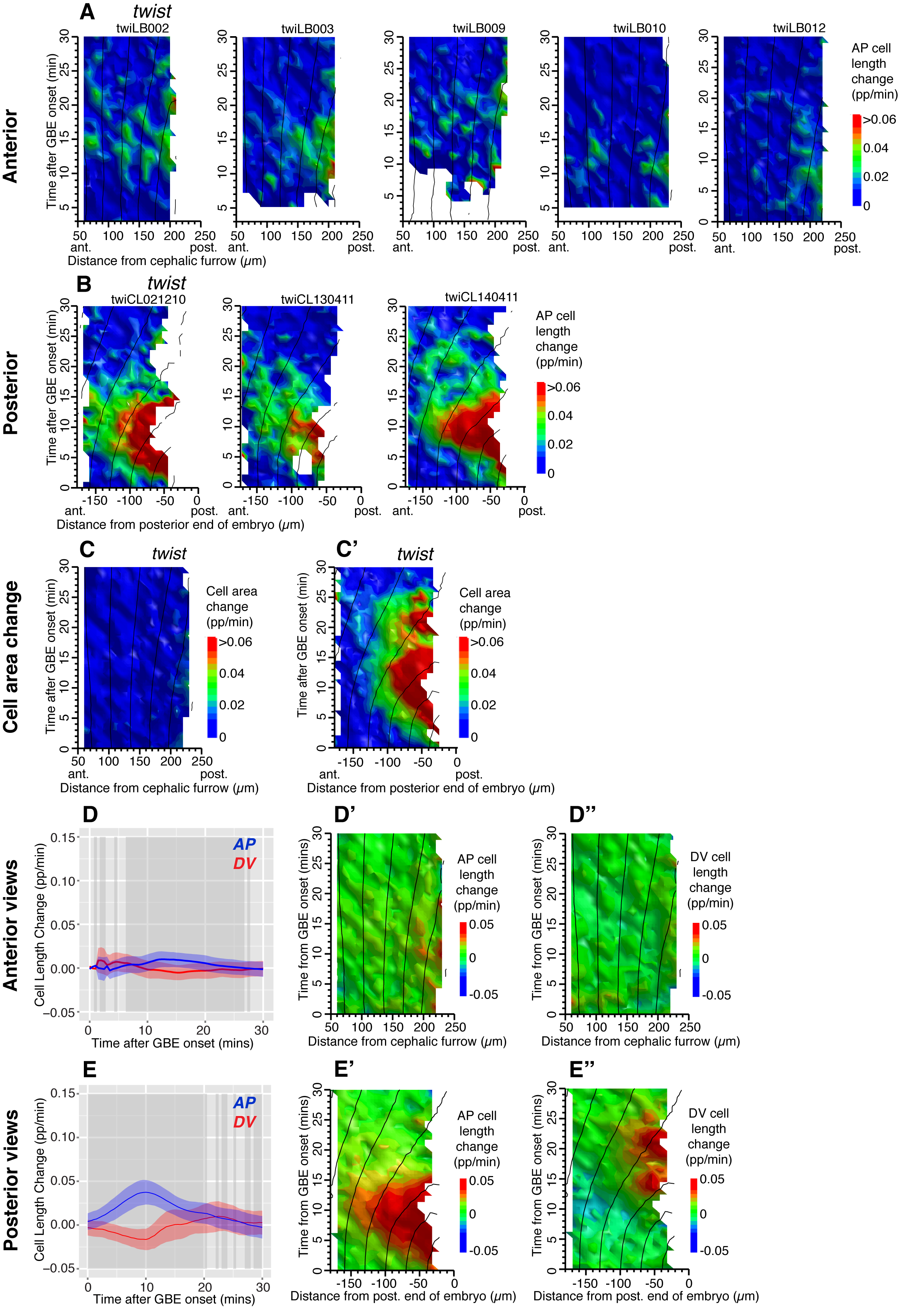

Supplement: S3 Fig — (A, B) Spatiotemporal maps summarizing AP cell length change over the first 30 min of GBE (y-axis) and as a function of cell position in the AP axis (x-axis) for twi mutant embryos, for anterior (A) and posterior views (B), for each movie collected per genotype. The position in the AP axis is measured from the anterior and posterior landmarks defined in Fig 1. Note that the cells analyzed for the anterior field of views do not include those deformed by the cephalic furrow (See wild-type example in Fig 1C and 1D). (C, C’) Spatiotemporal maps summarizing cell area change as a function of time after GBE onset (y-axis) and position in the AP axis, for anterior (C) and posterior views (C’), averaged for five and three twi mutant embryos, respectively. (D–E”) Comparison of AP and DV cell length change for anterior (D–D”) and posterior views (E–E”) for twi mutant embryos. (D, E) Graphs summarizing AP (blue) and DV (red) cell length change as a function of time after GBE onset (x-axis), for anterior and posterior views. (D’, E’) Spatiotemporal maps summarizing AP cell length change as a function of time after GBE onset (y-axis) and position in the AP axis, for anterior and posterior views. (D”, E”) Corresponding maps for DV cell length change. Data associated with this Figure can be found in S3 Data. (TIF) [file pbio.1002292.s009.tif]

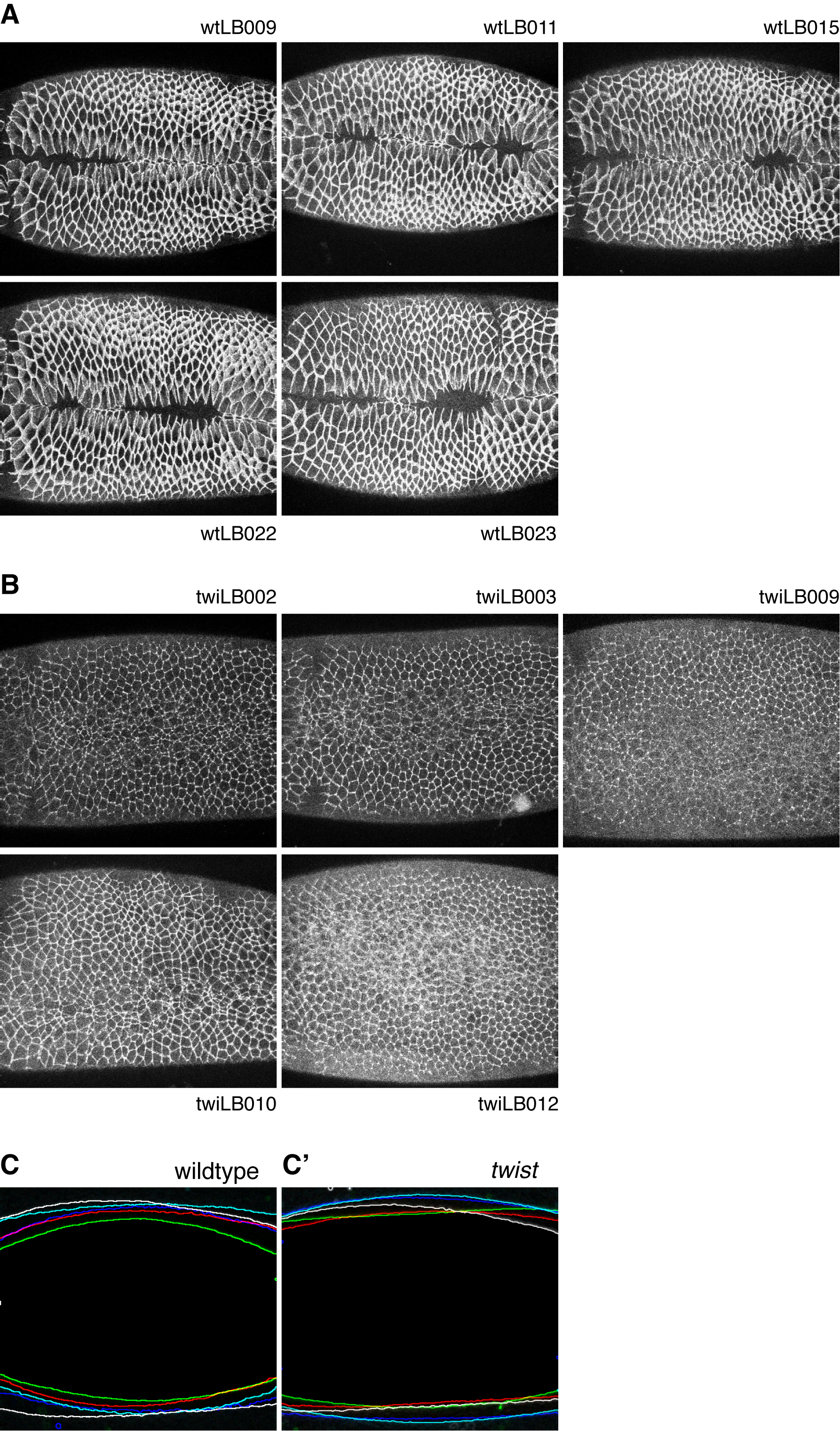

Supplement: S4 Fig — (A, B) Movie frames at timepoint 10 min after GBE onset for the anterior views, for wild-type (A) and twi mutant embryos (B). (C, C’) Drawn outlines of the five wild-type and five twi mutant embryos: the curvatures in twi embryos are less pronounced and the embryos wider compared to wild type. (TIF) [file pbio.1002292.s010.tif]

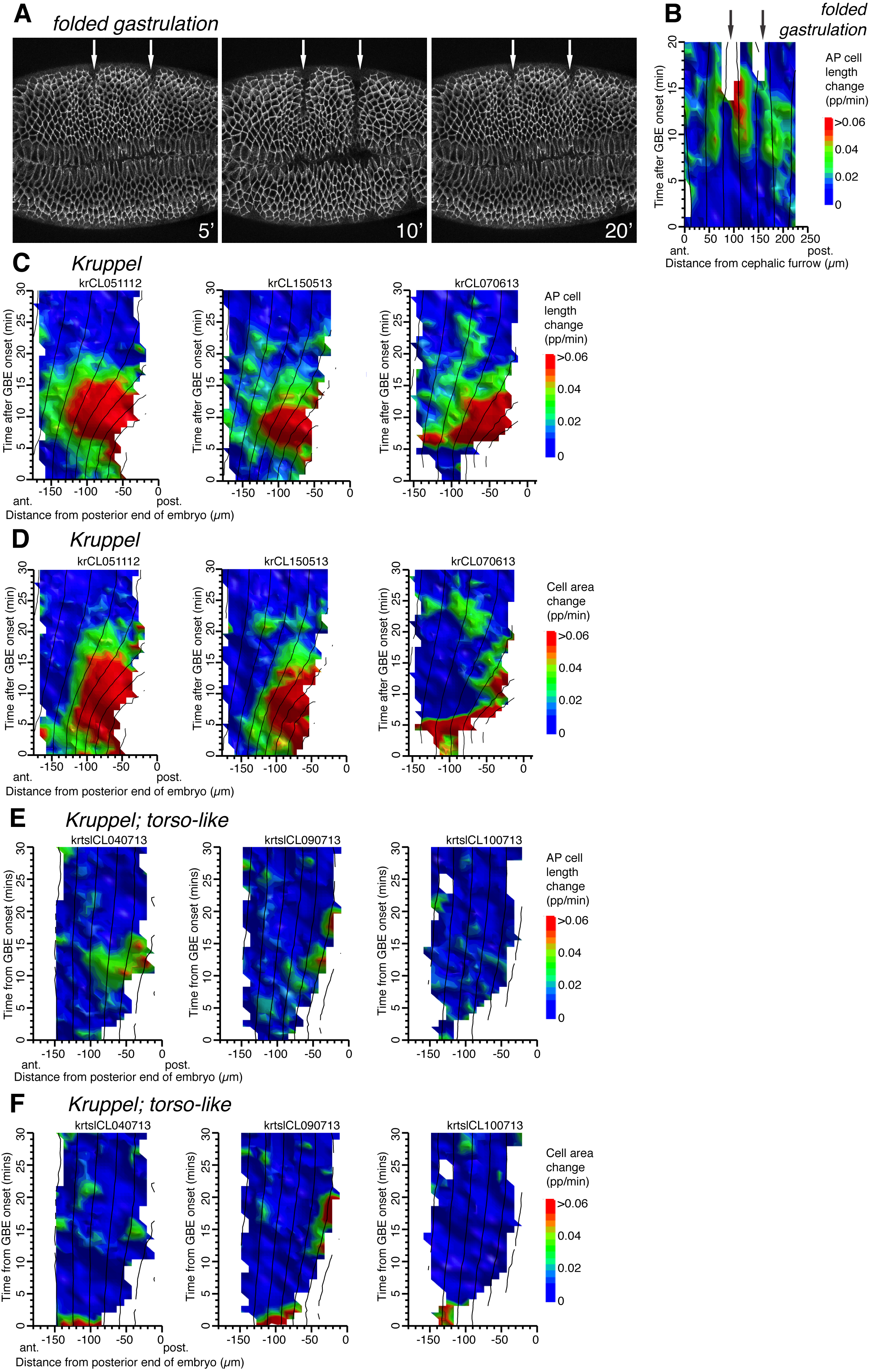

Supplement: S5 Fig — (A) Frames from a movie of a fog mutant embryo, at 5, 10, and 20 min after GBE onset. Folds start forming at ectopic sites early in axis extension. In this example, two deep folds form on one side of the embryo (arrows). (B) Corresponding spatiotemporal map summarizing AP cell length change over the first 20 mins of GBE (y-axis) and as a function of cell position in the AP axis (x-axis). The two folds seen in the movie are detected as AP cell length change on either side of the folds (indicated by arrows), from about 7 min onwards. There is no data available at the position of the folds, because the cells cannot be tracked. Note that outside the fold-induced signal, there is no obvious AP cell elongation gradient detectable in this mutant embryo. (C, E) Spatiotemporal maps summarizing AP cell length change over the first 30 mins of GBE (y-axis) and as a function of cell position in the AP axis (x-axis), for Kr (C) and Kr; tsl mutants (E), for each of the three movies collected per genotype. (D, F) Corresponding spatiotemporal maps summarizing cell area changes for each genotypes. (TIF) [file pbio.1002292.s011.tif]

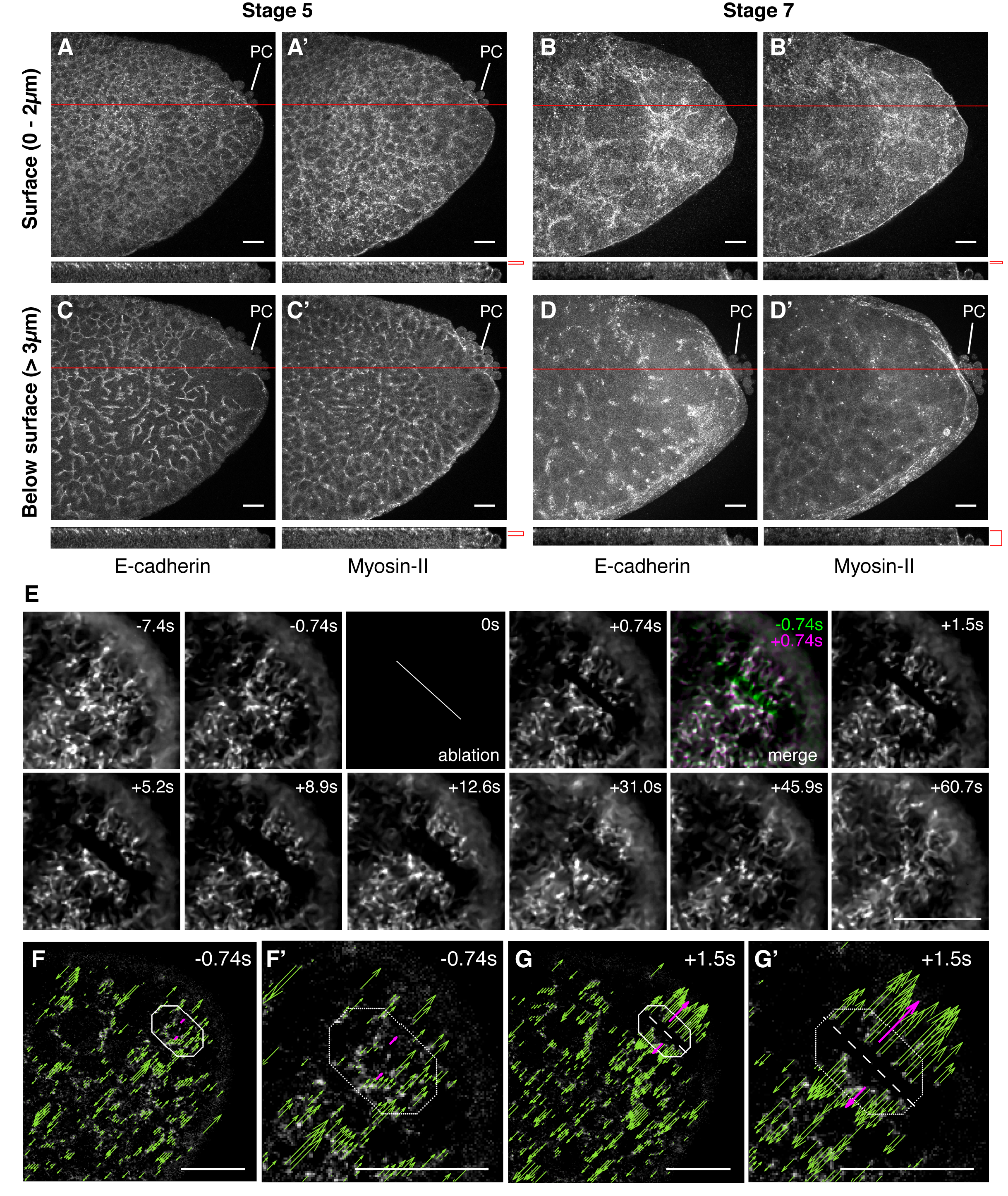

Supplement: S6 Fig — Scale bars are 20 microns for all panels. (A–D’) Posterior lateral views of fixed acellular embryos stained against E-cadherin and Myosin II (using antibody against mono-phosphorylated MRLC). Two stages are shown, just before gastrulation movements start (estimated stage five; A, A’, C, C’) and during gastrulation (estimated stage seven; B, B’, D, D’). For each stage, a projection of confocal sections shows the signal close to the surface (0–2 μm, A–B’) and a little deeper (> 3 μm, C–D’). The confocal sections used for each projection are shown by a red bracket in the reconstructed cross-section underneath each panel. The position of the cross-sections is indicated by a red line in each panel. PC are indicated. (E) Example of a laser ablation experiment for a DV-oriented cut at the posterior of an acellular embryo. Confocal images of the Myosin II signal are collected every 0.742 ms (timepoints displayed are indicated on panels) for 20 frames before and 120 frames after the cut (time zero, no image is acquired during ablation). Note that the images shown here are destriped and denoised (see supplementary Materials and Methods). The cut is seen as a gap in the Myosin II meshwork. The timepoints just before and after the cut are overlaid to show the displacement of the signal (merge). The gap caused by ablation continues to open for approximately 10–15 sec. Later on, new Myosin II signal moves in, eventually “repairing” the gap by about 1 min post-ablation. (F–G’) PIV analysis of Myosin II flows for the ablation experiment shown in E, overlayed on Myosin II signal (the images shown here are destriped but not denoised). The optical flows are represented with green arrows, which show displacement between the timepoint shown and the previous one, scaled by a factor of 25 (only 1 arrow in every 3 x 3 grid is visualized). Only the flow component perpendicular to the cut are visualized and analyzed. The velocity of optical flows are analyzed within a small region around eac [file pbio.1002292.s012.tif]
