# Supplementary material for: Mechanical Coupling between Endoderm Invagination and Axis Extension in Drosophila
Source: PLoS Biol. 2015 Nov 6;13(11):e1002292. doi: 10.1371/journal.pbio.1002292 (PMC4636290; doi:10.1371/journal.pbio.1002292)
Supplement: S1 Text — (DOCX) [file pbio.1002292.s021.docx]

**S1 Text: Supporting Materials and Methods**

**Embryos genotype and collection**

For apical cell surface movies, wild-type embryos were *w; ubi-DE-cad-GFP*, *twist* mutant embryos were *w; twist[1], ubi-DE-cad-GFP* and *Kruppel* mutants were *w; Kr[1], ubi-DE-cad-GFP*. To obtain embryos double mutant for *Kruppel* and *torso-like* (a maternal effect mutation), *w;* *Kr[1], ubi-DE-cad-GFP*; *tsl[4]/Df[3R]ED6076* females were crossed with *w; Kr[1], ubi-DE-cad-GFP* males*.* For whole embryo imaging, genotypes of wild-type embryos were *w; resille-GFP; spider GFP* and *twist* mutant embryos were *w; twist[1]; spider-GFP*. The genotype of acellular embryos was *Df(2L)dpp[s7-dp35] 21F1–3;22F1–2 (halo) Df(2L)Exel6016(slam) P{SUPor-P}CG42748^KG09309^(CG34137)/CyOsqh–GFP [*[*1*](#_ENREF_1)*]*. Embryos were collected on grape juice plates from flies raised at 25°C, and dechorionated in bleach prior to imaging. Staging is according to [[2](#_ENREF_2)]. Embryo survival and mutant phenotype was checked systematically by allowing the imaged embryos to develop at 25°C in a humid chamber until the end of embryogenesis. We checked that wild-type embryos hatched as live larvae. All above mutants die at the end of embryogenesis and their phenotype was checked by mounting cuticles in Hoyer's medium, except for acellular embryos, where homozygous mutants were identified by the presence of the *halo* phenotype.

**Apical cell imaging**

Embryos were mounted ventrally as previously in Voltalef oil [[3](#_ENREF_3)] and imaged with a x40/1.3NA oil immersion objective lens on a upright Nikon E1000 microscope coupled to Yokogawa CSU10 spinning disc confocal scanner. Illumination was with a Spectral Applied Research LMM5 laser module (491nm excitation). Images were captured with a Hamamatsu ImagEM EM-CCD camera driven by Volocity software (Perkin-Elmer). For all genotypes except acellular embryos, confocal stacks of 15-25µm (images separated by 1μm in z) captured the apices of the cells (some room was left above the embryo when the stack was set up to allow for minor movement of the embryo). For acellular embryos, stacks of 20-65µm depth were used to be able to image the pole cells as well as the surface. Stacks were acquired every 30 seconds for 1-1.5 hours. Movies were recorded at 20.5 1°C measured with a high-resolution thermometer (Checktemp1). Homozygous mutants are identifiable during imaging based on their morphogenetic phenotype, but the phenotypes of embryos (and their viability) were also further checked by letting the embryos develop until the end of embryogenesis or using the *halo* mutation (see above). When imaging acellular embryos, acquisition was started at the onset of gastrulation movements.

**Tracking of apical cell contours**

The confocal z-stacks were filtered to reduce noise (median and highpass or tophat) and segmented in ‘o’Tracks’ as described previously [[3](#_ENREF_3), [4](#_ENREF_4)]. Automatic tracking identified the majority of well imaged cells. Occasional misidentification was corrected manually in some movies by deleting mistracked cell membranes and by adding unidentified ones. Filters were also used to remove mistracked cells based on their absolute size or their change in size (very small or large cells were excluded, as were those that changed drastically in size from one frame to the next), their speed of movement from one frame to the next (in order to remove cells that are incorrectly linked in time), and the number of frames for which each cell exists. Threshold values used for filtering were determined for each movie, carefully choosing those values which excluded the majority of mistracked cells, but preserved as many well tracked cells as possible. Incomplete cells at the edge of the embryo were also removed prior to analysis.

**Analysis of apical cell deformation**

*Analysed cells.* We first selected carefully the cell populations to be analysed. In all movies, mesoderm and mesectodermal cells were excluded from analyses based on their dorso-ventral coordinates, taking into account the tissue translation during extension (using so-called comoving DV coordinates). In addition for anterior movies, cells of the head (if visible) and anterior 60µm of the trunk were excluded based on their absolute position in embryo (static co-ordinates). Figs 1D, D’; 2A, B; 3C, D; and 5E, F show movie frame examples of the cell populations analysed after exclusion of unwanted cells, for anterior and posterior movies (see also movies S1, S2, S5, S6).

*Cell shape strain rates.* To measure cell shape change, a best-fit ellipse is calculated for each cell shape at each time point. The change in shape of the cell ellipse over time is measured as orthogonal strain rates in units of proportional rates (pp/min) [[4](#_ENREF_4)] (see Fig 1B). Cell shape strain rates are then projected onto embryonic axes, AP or DV, and are called “AP or DV cell length change” in Figs and shortened to “AP or DV cell elongation” in the main text. Note that most of the data shown in this paper is AP cell length change, which when positive contributes to tissue extension along the AP axis of the embryo. Proportional rate of cell area change is calculated as the mean of the AP and DV cell length change. All colour-coded scales are 0-0.06 pp/min (the highest value of which indicate a 6% increase in cell length or area per minute). Strain rates calculated for each cell can be shown in movie frames as a colour coded dot in the center of a given cell (see for example movies S1, S2, S5, S6).

*Movie synchronization.* The number of embryos analysed for each experiment was: for anterior views, 5 for wt and 5 for *twi* [3]; for posterior views, 4 for wt, 3 for *twi* , 3 for *Kr* and 3 for *Kr; tsl*. To be able to average the data between embryos of the same genotype and to compare different genotypes, we synchronized the movies. The strategy for synchronization was the same as in [3], where we used the onset of germband extension as zero for all the movies. This was determined quantitatively by measuring total tissue strain rate in the direction of extension (this is a different measure from cell shape strain rate) [3,4]. Because tissue strain rates fluctuate around the onset of germ-band extension, we used a given threshold of tissue strain rate (in the AP axis) to synchronize the movies. The threshold was the same within a given genotype, but different between genotypes because their tissue strain rates differ. For anterior movies, the synchronization threshold is 0.01 pp/min for wild-type and 0.005 pp/min for *twist*- embryos [3]. For posterior movies, we used a synchronization threshold of 0.03 pp/min for wild type and *Kruppel*, 0.02 pp/min for *twist* and 0.01 pp/min for *Kruppel; torsolike.* Once all movies of the same genotype were synchronized, we calculated the average tissue extension strain rate over time for each genotype. Synchronisation between all movies was checked to be reasonable by comparing timings of key events such as mesoderm invagination and mesectodermal cell division. In the case of *Kruppel and Kruppel; torsolike,* the timings of these events were used to adjust the intergenotype synchronisation. This was necessary for *Kruppel* because for one movie (krCL070613), relatively few ectodermal cells are visible in the field of view in the first 5 minutes of germband extension, which affected the accuracy of the average tissue extension strain rate for this genotype at this time. It was necessary for *Kruppel; torsolike* because tracking around the start of germband extension was worse than normal for two of the movies (090713 and 100713), because of apparently lower expression of *ubi-DE-cad-GFP* in this genotype, which affected the accuracy of the average tissue extension strain rate and synchronization using the tissue threshold method.

*Data summaries.* Once all the movies were synchronized, we summarized data spatially and/or temporally. The contributions of cells to all strain rate summaries below are area-weighted. Graphs of cell shape strain rate plotted against time summarise data from all cells included in the analysis. Graphs of cell shape strain rate plotted against the AP axis show data for a specified timepoint or time period and summarise all data in DV. Spatial-temporal maps (contour plots) also summarise data in DV whilst plotting it against the orthogonal AP axis and time or summarise data in time whilst plotting it against the AP and DV axes. Distances along embryonic axes are given in µm from the ventral midline for the DV axis, µm from the cephalic furrow for the AP axis in anterior movies, and µm from the posterior end of the field of view for the AP axis in posterior movies. For the latter, because we do not image the whole depth of the embryo, this does not correspond to the very posterior tip of the embryo and the position of this landmark will thus depend upon the curvature of the embryo, which might be different between genotypes (see Fig 1A’ and discussion). Note that the black lines overlaid on spatiotemporal maps indicate tissue translation.

*Statistics.* To test for evidence of differences between data derived from different embryos, we used the mixed-effects model constructed for [3]. Briefly, we estimated the P-value associated with a fixed effect of differences between genotypes, allowing for random effects contributed by differences between embryos within a given genotype, calculated at each time point or AP location. Ribbons are drawn for the whole span of analysis for control embryos (coloured blue) and for test embryos (coloured red). For spatial data, continuous data were binned into 100 equally-spaced bins along the abscissa, for plotting and statistical tests. For the ribbons only, the mean trends and ribbon width are calculated from data averaged to reduce noise: a box average of +/- three bins along the abscissa were used. The widths of ribbons straddling average strain rates represent a standard error calculated from the sums of within-experiment variance and between experiment variance. To test where test embryos were significantly different (P < 0.05) from wild type, mixed-model was applied, with embryo as the random variable. No data averaging, other than binning, was used. The regions where P < 0.05 are depicted with a grey-shaded box.

**Whole embryo imaging**

Embryos were washed in PBS-Tween and mounted vertically in 1.5% low melting point agarose (Sigma) containing 0.5µm ‘yellow’ estopor microspheres (Merck) at a concentration of 1/1000 in a cylinder using glass capillaries (Brand). The capillary was attached to a rotation motor and immersed in a chamber filled with PBS, where it was imaged using mSPIM at a room temperature of 28-30°C. Images were collected every 3 µm along the *z*-axis, from the surface of the embryo to just past its centre using a 20x/0.5NA water dipping objective lens (Leica) and an EM-CCD camera (Andor). The illumination arms were composed of Coherent Sapphire LP lasers (100 mW, 488nm), 1-kHz resonant mirrors, cylindrical lenses and two Zeiss 10×/0.2NA air illumination objectives. The embryo was illuminated perpendicular to the angle of acquisition; each image was taken first with the embryo illuminated from one side and then the other. The embryo was imaged from four angles, separated by a 90° rotation, so that all the cells of the embryo were imaged. The entire embryo was imaged every 30 seconds for 60 minutes. The data from the four views were reconstructed post-acquisition into a single image stack, using bead-based registration and content based fusion (Fiji plugin) [[5](#_ENREF_5)]. The imaging process was confirmed to have no adverse effect on development (see above).

**Temporal mapping in whole embryo movies**

Reconstructed movies of three wild-type and three *twist* mutants were viewed in 4D in custom software (Browser and Tracer) written in Interactive Data Language (IDL, Exelis) [[6](#_ENREF_6)] and morphogenetic movements were identified by eye. The beginning of germband extension defined as the first timepoint with detectable posteriorward displacement of ventral cells (see Fig 4C, C’). The beginning of mitoses in the head was defined as the timepoint in which the first cytokinesis event in the head was observed (see Fig 4D-D”). The beginning of posterior endoderm invagination was defined as the first timepoint in which the apices of the posterior endoderm cells shrank detectably, and then continued to shrink in subsequent frames (see Fig 4E, E’). The beginning of mesodermal tube sealing was defined as the timepoint when the right and left sides of the tissue first met to begin forming the internal mesodermal tube (see Fig 4E, E’). The beginning of dorsal fold formation was defined as the first timepoint at which detectable buckling (ie basalward movement of cell apices) could be seen on the dorsal side of the embryo (see Fig 4G, G’). The beginning of dorsal contraction was defined as the first timepoint at which dorsal anterior and dorsal posterior cells could be detected moving towards each other (see Fig 4H, H’).

**Antibody stainings of acellular embryos**

We followed standard methods as in [[7](#_ENREF_7)] for fixing and staining acellular embryos, using the primary antibodies anti-DE-Cadherin (1/50, DCAD2, developed by T. Uemura, Developmental Studies Hybridoma Bank) and anti-Sqh1P (1/100, a gift from R. Ward,[[8](#_ENREF_8)]).

**Particle Image Velocimetry of Myosin II flows in acellular embryos**

Acellular embryos expressing *sqh-GFP* to label Myosin II were selected during cellularisation based on their *halo* phenotype, mounted laterally and imaged as described above. A plugin in FIJI was used to perform Particle Image Velocimetry (PIV) of images of acellular embryos [[9](#_ENREF_9)]. Stacks were processed to maximum-intensity Z-projections, and background signal from outside the embryo was removed manually from the images. Three iterations of PIV were run using the normalized correlation coefficient method, with interrogation window halving each time, giving a grid spacing of 8.7 × 8.7 µm in the plotted displacement field.

**Laser ablation of acellular embryos**

Mounting acellular embryos as above, laser ablation experiments were performed using a TriM Scope II Upright 2-photon Scanning Fluorescence Microscope controlled by Inspector Pro software (LaVision Biotec). The laser source for the microscope was a tuneable near-infrared (NIR) laser delivering 120 femtosecond pulses with a repetition rate of 80 MHz (Insight DeepSee, Spectra-Physics). The laser was tuned to 927nm, with an average power of 1.7 W. The maximum laser power allowed to reach the sample was set to 190 mW and an Electro-Optical Modulator (EOM) was used to allow microsecond switching between imaging and treatment laser powers. The laser light was focused by a 25x, 1.05 Numerical Aperture (NA) water immersion objective lens with a 2mm working distance (XLPLN25XWMP2, Olympus). Given the non-linearity of the 2-photon absorption process an NA of 1.05 allows incident laser light of 927nm wavelength to be focused to a spot with FWHM of 340nm laterally and 1.2µm axially, although the spatial extent of treatment at high powers will likely exceed this volume. Images were collected every 0.742ms for 20 frames before the ablation and 120 frames after the ablation, using a GaAsP photomultiplier tube.

Targeted line ablations of 20µm length were performed on the apical Myosin II mesh (approximately 2µm below the vitelline membrane) using a treatment power of 190 mW. Ablations were performed during image acquisition (with a dwell time of 10µsec per pixel), with the laser power switching between treatment and imaging powers as the laser was raster scanned across the sample. Line ablations were oriented parallel to the dorso-ventral embryonic axis (DV ablations) or parallel to the anterior-posterior embryonic axes (AP ablations) and were performed both near the posterior tip of the embryo (posterior ablations) or near the middle of the anterior-posterior axis of the embryo (anterior ablations).

**Analysis of recoil velocities from laser ablations**

In order to quantify recoil, we had to automatically detect the position of the ablation line in the images, choose a region of interest around it, detect the fluorescent structures inside this region and infer their motion from the video sequences. The region of interest in each movie is defined as a 20 pixel area on each side of the cut. The position of the cut is determined by thresholding the first image acquired after the cut (timepoint 21, +0.742 seconds) with a threshold estimated from a Gaussian model of pixel intensities. The Myosin II signal appears in the image as small fluorescent bright structures on dark background. The bright pixels are considered true signal if the à trous wavelet coefficients are deemed significant at two consecutive levels by a selection algorithm based on the control of the false discovery rate [[10](#_ENREF_10)]. Only embryos with more than 100 significant pixels of signal in the region of interest were included in the study. We first performed two pre-processing steps: a frequency filter to remove the stripes caused by electrical interference in the detector and a patch based denoising step [[11](#_ENREF_11)]. Subsequently, we computed the optical flow in order to estimate the velocity of the structures using the Lucas-Kanade algorithm [[12](#_ENREF_12)], on the significant pixels of signal in the region of interest for each embryo. Square windows of 12 pixels width were used in the computation of the optical flow. In order to use the flow of Myosin II to measure recoil away from the cut, only the velocity component of flow perpendicular to the cut is considered in the analysis. To take account of translation, the velocity at time point 22 (+ 1.484 seconds) was corrected by subtracting the average speed in the region of interest before ablation, computed from the optical flow of the three frames before the cut (timepoints 18, 19 and 20, -2.226, -1.484 and – 0.742 seconds) to give the normalised relaxation speed. For each side of the cut, the average normalised relaxation speed is computed and the two averages are combined (weighted average, taking account of the number of pixels of significant signal on each side of the cut) to give a measure of motion away from the cut (final normalised relaxation speed shown in Fig 6E). This final velocity was compared for the four conditions and a two sample t-test was used to look for statistically significant differences between groups (Fig 6E).

1. He B, Doubrovinski K, Polyakov O, Wieschaus E. Apical constriction drives tissue-scale hydrodynamic flow to mediate cell elongation. Nature. 2014;508(7496):392-6. Epub 2014/03/05. doi: 10.1038/nature13070. PubMed PMID: 24590071; PubMed Central PMCID: PMC4111109.

2. Wieschaus E, Nusslein-Volhard C. Looking at embryos. In: Roberts DB, editor. Drosophila, a practical approach. United States: Oxford University Press Inc., New York; 1998.

3. Butler LC, Blanchard GB, Kabla AJ, Lawrence NJ, Welchman DP, Mahadevan L, et al. Cell shape changes indicate a role for extrinsic tensile forces in Drosophila germ-band extension. Nature cell biology. 2009;11(7):859-64. Epub 2009/06/09. doi: 10.1038/ncb1894. PubMed PMID: 19503074.

4. Blanchard GB, Kabla AJ, Schultz NL, Butler LC, Sanson B, Gorfinkiel N, et al. Tissue tectonics: morphogenetic strain rates, cell shape change and intercalation. Nature methods. 2009;6(6):458-64. Epub 2009/05/05. doi: 10.1038/nmeth.1327. PubMed PMID: 19412170.

5. Preibisch S, Saalfeld S, Schindelin J, Tomancak P. Software for bead-based registration of selective plane illumination microscopy data. Nature methods. 2010;7(6):418-9. Epub 2010/05/29. doi: 10.1038/nmeth0610-418. PubMed PMID: 20508634.

6. England SJ, Blanchard GB, Mahadevan L, Adams RJ. A dynamic fate map of the forebrain shows how vertebrate eyes form and explains two causes of cyclopia. Development. 2006;133(23):4613-7. Epub 2006/11/03. doi: 10.1242/dev.02678. PubMed PMID: 17079266.

7. Lye CM, Naylor HW, Sanson B. Subcellular localisations of the CPTI collection of YFP-tagged proteins in Drosophila embryos. Development. 2014;In press.

8. Zhang L, Ward REt. Distinct tissue distributions and subcellular localizations of differently phosphorylated forms of the myosin regulatory light chain in Drosophila. Gene expression patterns : GEP. 2011;11(1-2):93-104. Epub 2010/10/06. doi: 10.1016/j.gep.2010.09.008. PubMed PMID: 20920606; PubMed Central PMCID: PMC3025304.

9. Tseng Q, Duchemin-Pelletier E, Deshiere A, Balland M, Guillou H, Filhol O, et al. Spatial organization of the extracellular matrix regulates cell-cell junction positioning. Proceedings of the National Academy of Sciences of the United States of America. 2012;109(5):1506-11. Epub 2012/02/07. doi: 10.1073/pnas.1106377109. PubMed PMID: 22307605; PubMed Central PMCID: PMC3277177.

10. Muresan L, Jacak J, Klement EP, Hesse J, Schutz GJ. Microarray analysis at single-molecule resolution. IEEE transactions on nanobioscience. 2010;9(1):51-8. Epub 2010/02/04. doi: 10.1109/TNB.2010.2040627. PubMed PMID: 20123580; PubMed Central PMCID: PMC2912528.

11. Boulanger J, Kervrann C, Bouthemy P, Elbau P, Sibarita JB, Salamero J. Patch-based nonlocal functional for denoising fluorescence microscopy image sequences. IEEE transactions on medical imaging. 2010;29(2):442-54. Epub 2009/11/11. doi: 10.1109/TMI.2009.2033991. PubMed PMID: 19900849.

12. Lucas DB, Kanade T, editors. An Iterative Image Registration Technique with an Application to Stereo Vision. International Joint Conference on Artificial Intelligence; 1981.
